# Spatial equity in the allocation of lifesaving resources: a cross-sectional spatial evaluation of automated external defibrillators in four first-tier Chinese cities

**DOI:** 10.1186/s12942-026-00470-w

**Published:** 2026-05-02

**Authors:** Aiping Gou, Lei Wang, Jiangbo Wang, Chunyan Gou, Jing Li

**Affiliations:** 1 College of Ecological Technology and Engineering, Shanghai Institute of Technology, Shanghai, 201418 China; 2 College of Architecture, Nanjing Tech University, Nanjing, 211816 China; 3 Chongqing Traditional Chinese Medicine Hospital, Chongqing, 400021 China; 4 Department of Acupuncture, Yueyang Hospital of Integrated Traditional Chinese and Western Medicine, Shanghai University of Traditional Chinese Medicine, Shanghai, 200437 China

**Keywords:** Automated external defibrillator (AED), Spatial equity, Comprehensive evaluation model, Geographically Weighted Regression (GWR), Two-Step Floating Catchment Area (2SFCA)

## Abstract

**Background:**

The timeliness of treatment for out-of-hospital cardiac arrest (OHCA) is critical for patient survival. Automated External Defibrillators (AEDs) are a proven effective intervention, yet China’s rapidly developing Public Access Defibrillation (PAD) program may be accompanied by significant spatial inequities in AED distribution.

**Methods:**

This study developed a comprehensive multi-dimensional evaluation model to assess the spatial equity of AED allocation in four first-tier Chinese cities: Beijing, Shanghai, Guangzhou, and Shenzhen. The model integrated four dimensions: resource allocation (supply–demand ratio), spatial coverage (service coverage index), opportunity accessibility (accessibility index via an enhanced Gaussian two-step floating catchment area method), and spatial distribution (Gini coefficient). These dimensions were aggregated into a Comprehensive Equity Index (CEI) using the Entropy Weight Method (EWM). Leveraging high-resolution gridded population data and precise AED locations, our analysis captures fine-scale spatial variations often obscured in aggregate statistics. Furthermore, to uncover the spatially heterogeneous drivers of equity, we employed an integrated Principal Component Analysis and Geographically Weighted Regression (PCA-GWR) framework to analyze socioeconomic and urban environmental factors.

**Results:**

The results indicate that: (1) Overall comprehensive equity was low across all cities (mean CEI < 0.3). Shenzhen exhibited the highest equity (mean CEI: 0.252), followed by Beijing (0.207), with Shanghai and Guangzhou lagging. (2) A significant “core-periphery” disparity was observed in all cities, with core districts showing markedly higher equity than suburban districts, a gap particularly pronounced in Beijing and Shanghai. (3) The PCA-GWR analysis revealed pronounced spatial heterogeneity in the associations between external factors and AED equity. Degree of urbanization showed a generally positive association, which was consistently weaker in urban cores. Public service facility provision exhibited inconsistent (often negative) associations, while the wealth-population density trade-off demonstrated marked city-specific variation.

**Conclusions:**

This study provides a systematic, multidimensional assessment of AED allocation equity in major Chinese cities. By employing a spatially nuanced PCA-GWR framework, it reveals that equity is shaped by complex, location-specific interactions of urban development, service provision, and socioeconomic structure. The findings underscore the necessity for spatially differentiated policy interventions within China’s PAD program to achieve more equitable and efficient deployment of these lifesaving resources.

**Supplementary Information:**

The online version contains supplementary material available at 10.1186/s12942-026-00470-w.

## Introduction

Out-of-hospital cardiac arrest (OHCA) is regarded as a leading cause of death worldwide due to its high incidence, low survival rate, and unpredictability, and has attracted increasing attention in recent years [1]. A study published in *The Lancet* reported an OHCA incidence of 95.7 per 100,000 people in China, compared to 84 per 100,000 in European countries. Owing to China’s large population base, the absolute number of OHCA cases far exceeds the global average [2, 3]. More concerning is the survival outcome: while the global average survival rate ranges from 5 to 10%, China’s is only 1.3% [4, 5]. This disparity, largely attributable to pre-hospital care bottlenecks such as delayed Emergency Medical Services (EMS) response, low bystander intervention rates, and critically, the insufficient availability and use of Automated External Defibrillators (AEDs) [4, 5], underscores the critical importance of timely on-site intervention. In OHCA emergency response, the effectiveness of conventional cardiopulmonary resuscitation (CPR) is highly dependent on the knowledge and skills of the responder, and studies indicate significant geographic disparities in such knowledge and implementation rates, particularly between urban and peri-urban/rural populations [6]. In contrast, the AED is a standardized device that is also operable by laypersons. It works by restoring effective heart rhythm through electrical defibrillation of shockable rhythms such as ventricular fibrillation [7]. More importantly, existing evidence indicates that prompt use of AEDs can increase survival rates to 50–70% [7]. This underscores that AED deployment is a crucial measure for improving OHCA survival rates. The current status of public access defibrillation (PAD) is suboptimal globally, with bystander defibrillation rates reported at only 2–4% [8]. In contrast, studies in Shenzhen, China, report a much lower rate of 0.2% [9]. Given this context, China’s PAD program started relatively late but has developed rapidly [10]. In 2020, the *Consensus statement on layout and delivery of automatic external defibrillator in China* was issued [11], followed by the *Guidelines for the Configuration of Automated External Defibrillators in Public Places (Trial)* in 2021 [12], accelerating the national PAD effort. However, rapid expansion has also introduced challenges. Significant spatial mismatches exist in AED placement, leading to inadequate coverage in high-density or vulnerable population areas [13]. Moreover, issues such as unclear signage and poor management reduce practical public access to AEDs, contrary to policy intentions [14]. These spatial and institutional barriers jointly contribute to inequitable access to emergency resources across social groups, constituting unfairness in public health resource allocation [15]. It is important to emphasize that such “facility inequality” not only affects individual survival chances but also raises issues of social justice, relating directly to United Nations Sustainable Development Goal 10 (Reduced Inequalities) [16]. This study therefore aims to systematically evaluate the spatial equity of AED distribution in major Chinese cities. Specifically, it addresses three questions: (1) What is the overall level of spatial equity in AED allocation? (2) How do equity levels vary across regions, particularly between urban cores and suburbs? (3) What external factors are associated with these equity patterns?

Spatial configuration of AED facilities involves studying the distribution, coverage, accessibility, and other spatial aspects of AEDs to identify deficiencies and optimize placement, thereby increasing defibrillation efficiency and OHCA survival rates. Existing research on AED spatial configuration includes studies on accessibility [17], service coverage [18], location selection [19], and indoor placement [20], using methods such as surveys [14], expert evaluation [21], mathematical modeling [22], spatial analysis [23], and statistical methods [24]. AED spatial research can be categorized into three themes: (1) Spatial pattern and coverage analysis, often using geographic information systems (GIS) and network analysis to examine AED distribution and service areas [25–27]; (2) Facility location optimization: These studies integrate geospatial models with mathematical optimization algorithms to refine current AED placements under resource constraints, aiming to achieve utility maximization objectives, such as maximum geographic coverage or maximum population coverage [28–30]. (3) Facility accessibility: These studies frequently employ methods such as the gravity model [31], minimum nearest distance [32], and the two-step floating catchment area (2SFCA) method [33], which incorporate the three core aspects of supply, demand, and spatial impedance (i.e., travel cost), to evaluate people’s accessibility to facilities. Previous studies have contributed significantly to understanding AED distribution and enhancing OHCA response efficiency.

Building on this foundation, recent international research (2021–2025) has yielded new findings that vary considerably across countries. In Sweden, Schierbeck et al. (2021) demonstrated that 61 AED-equipped drones deployed in high-incidence areas could cover 25.7% of out-of-hospital cardiac arrests (OHCAs) within 8 min, offering a solution for remote regions [34]. In Switzerland, Aeby et al. (2021) proposed a pragmatic relocation strategy—identifying “hotspots” and “overlays”—showing that adding 40 AEDs and relocating 17 existing ones could increase coverage from 7.5% to 17.6% [35]. In Great Britain, Burgoine et al. (2024) found that the most deprived neighborhoods had significantly longer distances to around-the-clock accessible AEDs (by 99–317 m), revealing an “out-of-hours” equity gap [36]. In Brazil, Bitencourt et al. (2025) integrated ambulance delay zones with a maximal coverage model, showing that 100 optimally placed AEDs could reduce median defibrillation time from 11.6 to 5.3 min [37]. In South Korea, Oh et al. (2021) identified a stark mismatch between AED quantity and utilization: nationally, only 0.38% of installed AEDs were used annually, and legally mandated venues accounted for 56.7% of AEDs but had a utilization rate one-third that of non-mandated venues [38]. In Washington D.C., Zhang et al. (2023) utilized mobile phone data to develop a spatiotemporal optimization model that captures hourly population mobility, revealing that a configuration optimized for off-peak hours achieved better 24-h average performance than one optimized for peak hours [39]. In Hangzhou, China, Chen et al. (2023) proposed a multi-criteria risk assessment framework that integrates land use, vulnerable population distribution, and traffic accessibility, identifying a three-phase deployment plan in the absence of historical OHCA records [40]. Collectively, these studies underscore the importance of dynamic demand, socioeconomic equity, and utilization efficiency, while also highlighting the value of spatially explicit, multi-dimensional methodological frameworks for optimizing AED allocation.

Despite these advances, however, few studies have explicitly addressed the multidimensional nature of equity in AED allocation. “Equity” is a multidimensional concept, and spatial equity is widely recognized as having multiple facets, as a single indicator is insufficient to fully capture the complexity of equitable geographic allocation [41]. In the context of lifesaving public facilities, spatial equity refers to the fair distribution of resources—ensuring that all residents, regardless of where they live, have reasonable and non-discriminatory access to essential emergency services within acceptable temporal and spatial limits [42]. Existing equity research frequently draws on John Rawls’ theoretical framework of justice [43, 44]. Rawls’ “veil of ignorance” thought experiment posits that a just society should uphold: (1) equal basic liberties for all; (2) fair equality of opportunity for social positions; and (3) the difference principle, which permits social and economic inequalities only insofar as they benefit the least advantaged members of society [45, 46]. In the context of spatial resource allocation, this implies that the geographic distribution of facilities should not systematically disadvantage any group based on residential location, and allocation mechanisms should not exacerbate structural inequalities. Accordingly, equity in AED allocation means that all societal members have equal opportunity to access AED services. Current approaches to medical facility equity include statistical analysis [47], spatial coverage [18], the Gini coefficient [48], Theil index [49], location entropy [50], and accessibility measures [51]. For example, Li et al. (2022) evaluated healthcare institution equity in Guangzhou from population and geographical dimensions via the Gini coefficient; they found inequities existed in both, particularly severe geographical inequity for all institution levels [52]. Su et al. (2024) analyzed multilevel healthcare facilities in Shanghai and found inequitable access between central and suburban areas, especially for tertiary hospitals [53]. Li et al. (2023) reported a high-demand-low-supply pattern in suburban Tianjin, with rising inequity risk as demand grows in suburban and rural areas [54]. Such studies on medical resource equity have greatly informed health policy and spatial justice.

Despite these contributions, prior research on AED spatial equity exhibits several limitations that this study aims to address. Firstly, there are data constraints: early studies often relied on small samples due to underdeveloped AED registries, likely underestimating true deployment density. Secondly, geographical scope: most studies focused on single cities or urban cores, lacking comparative analysis across cities or urban–rural gradients, which limits understanding of equity patterns across development stages. Thirdly, limited assessment dimensions: many studies adopted a one-dimensional approach (e.g., coverage or accessibility alone), whereas a comprehensive multi-dimensional equity assessment is needed to reflect real-world social disparities in AED access.

To bridge these gaps, this study conducts a spatially explicit and multidimensional assessment of AED allocation equity, with a comparative focus across major urban contexts. We select Beijing, Shanghai, Guangzhou, and Shenzhen as our study areas—four first-tier Chinese cities that have systematically implemented PAD programs and publicly released AED location data [11]. Their representativeness addresses the first two limitations by enabling robust cross-city comparison. Methodologically, we develop a comprehensive framework integrating four complementary dimensions of equity, countering the third limitation of one-dimensional assessments. These dimensions are aggregated into a Comprehensive Equity Index (CEI) for integrated comparison. Furthermore, to uncover the spatially heterogeneous mechanisms driving equity patterns, we employ an integrated Principal Component Analysis and Geographically Weighted Regression (PCA-GWR) model. The research contributes in two ways: (1) Empirically, this study provides a comparative analysis of multidimensional equity in four first-tier Chinese cities at varying stages of PAD implementation, moving beyond single-city or single-metric assessments to reveal how the structure of equity differs across urban contexts. (2) Methodologically, it contributes by developing and integrating a four-dimensional equity evaluation framework with a PCA-GWR model. This approach not only overcomes the limitations of one-dimensional assessments but also uniquely captures the spatially heterogeneous mechanisms driving equity, offering a novel lens for identifying locally specific determinants. (3) From a data perspective, this study demonstrates the critical importance of leveraging high-resolution, open-source geospatial data (e.g., WorldPop, OpenStreetMap, Points of Interest) for precisely uncovering fine-grained spatial inequities in public health resource allocation. By moving beyond conventional administrative boundary-based analyses, our approach captures nuanced local variations that are essential for formulating precisely targeted spatial policies and evidence-based interventions, thereby enhancing the accuracy and relevance of research findings for policy making. The results not only offer a new methodological framework for health resource equity evaluation but also provide evidence-based guidance for other cities to achieve equitable AED allocation, extending empirical research on equity in public health resources from a spatial justice perspective.

## Materials and methodology

### Study area

**Fig. 1:**
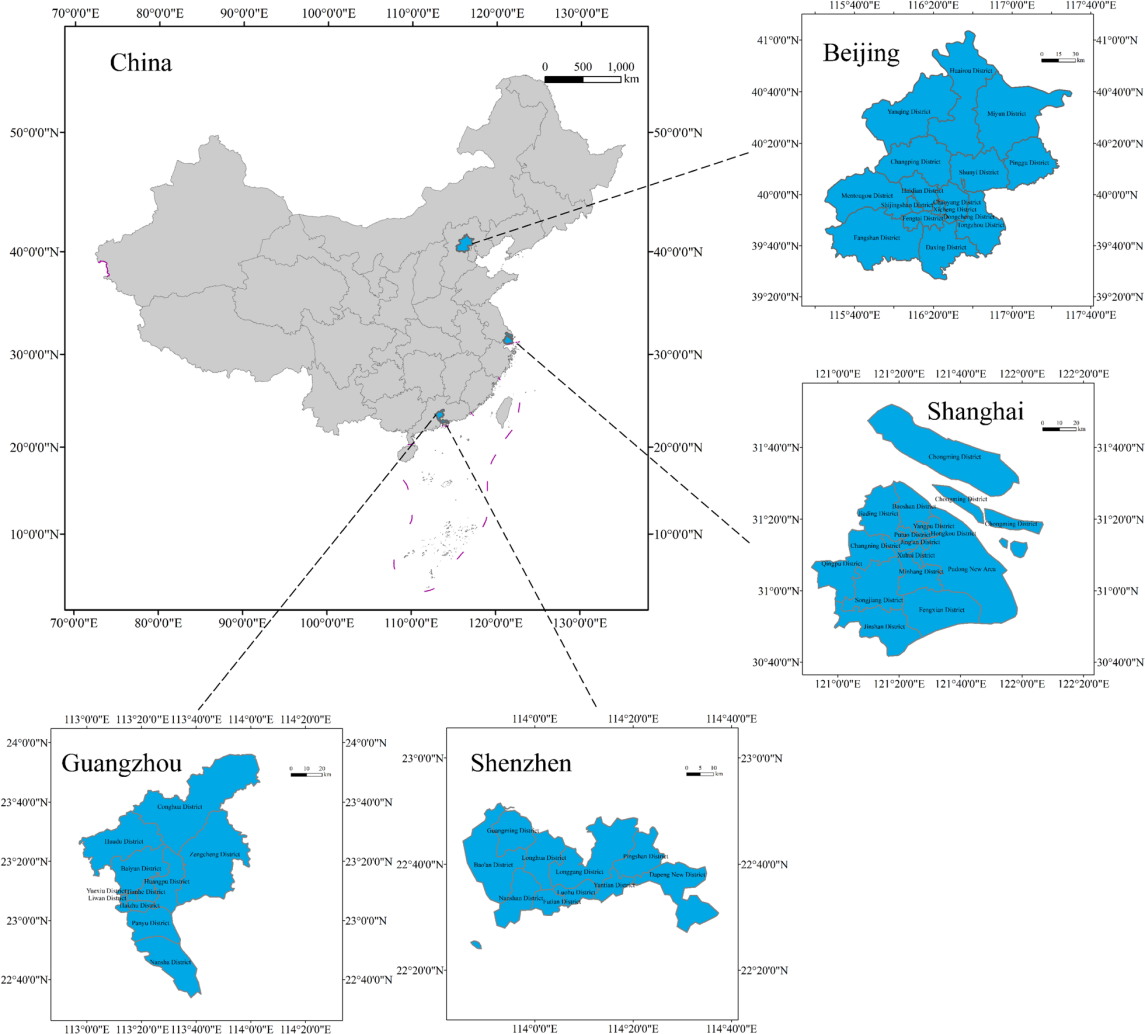
Location of the study areas: Beijing, Shanghai, Guangzhou, and Shenzhen. Image source: Map drawn by the author based on Survey Numbers GS(2024)0650. Due to scale limitations, district labels in the core areas may be crowded and less legible; high-resolution maps with numbered districts and a corresponding name list are provided in Supplementary Material S1 (Fig. S1-S4) for detailed reference

Beijing, Shanghai, Guangzhou, and Shenzhen are China’s four major first-tier cities (Table 1, Fig. 1), leading in comprehensive strength and competitiveness among mainland Chinese cities. They were also among the first to propose AED deployment plans and currently exhibit relatively good facility penetration, making them highly representative for this study. For the purpose of this study, we operationalize the urban core and suburban areas at two geographical scales: the district level and the subdistrict level. The terms ‘core districts’ and ‘suburban districts’ refer to the broader administrative divisions of each city, while ‘core subdistricts’ and ‘suburban subdistricts’ refer to the finer-grained subdistrict units within those districts, as classified in (Table 2).

**Table 1:** General situation of the study areas

City	Status	Administrative divisions	Area (km^2^)	Population (10,000 persons 2022)	GDP (100 million RMB, 2022)
Beijing	Capita, megacity	16	16,410.54	2184.30	41,610.90
Shanghai	Municipality, megacity	16	6340.50	2475.89	44,652.80
Guangzhou	Provincial capital, megacity	11	7434.40	1873.41	28,839.00
Shenzhen	special Economic zone, megacity	10	1997.47	1766.18	32,387.68

**Table 2:** Administrative division hierarchy

City	Zone type	Number of districts	Number of subdistricts
Beijing	Core	6	134
	Suburban	10	197
Shanghai	Core	7*	95
	Suburban	9*	119
Guangzhou	Core	4	79
	Suburban	7	91
Shenzhen	Core	3	28
	Suburban	7	46

The asterisk (*) for Shanghai indicates that the classification is based on the *Shanghai City Master Plan (2017–2035)*, where subdistricts within the outer ring road of Pudong New Area are classified as core districts, and those outside are classified as suburban districts

### Data

**Fig. 2:**
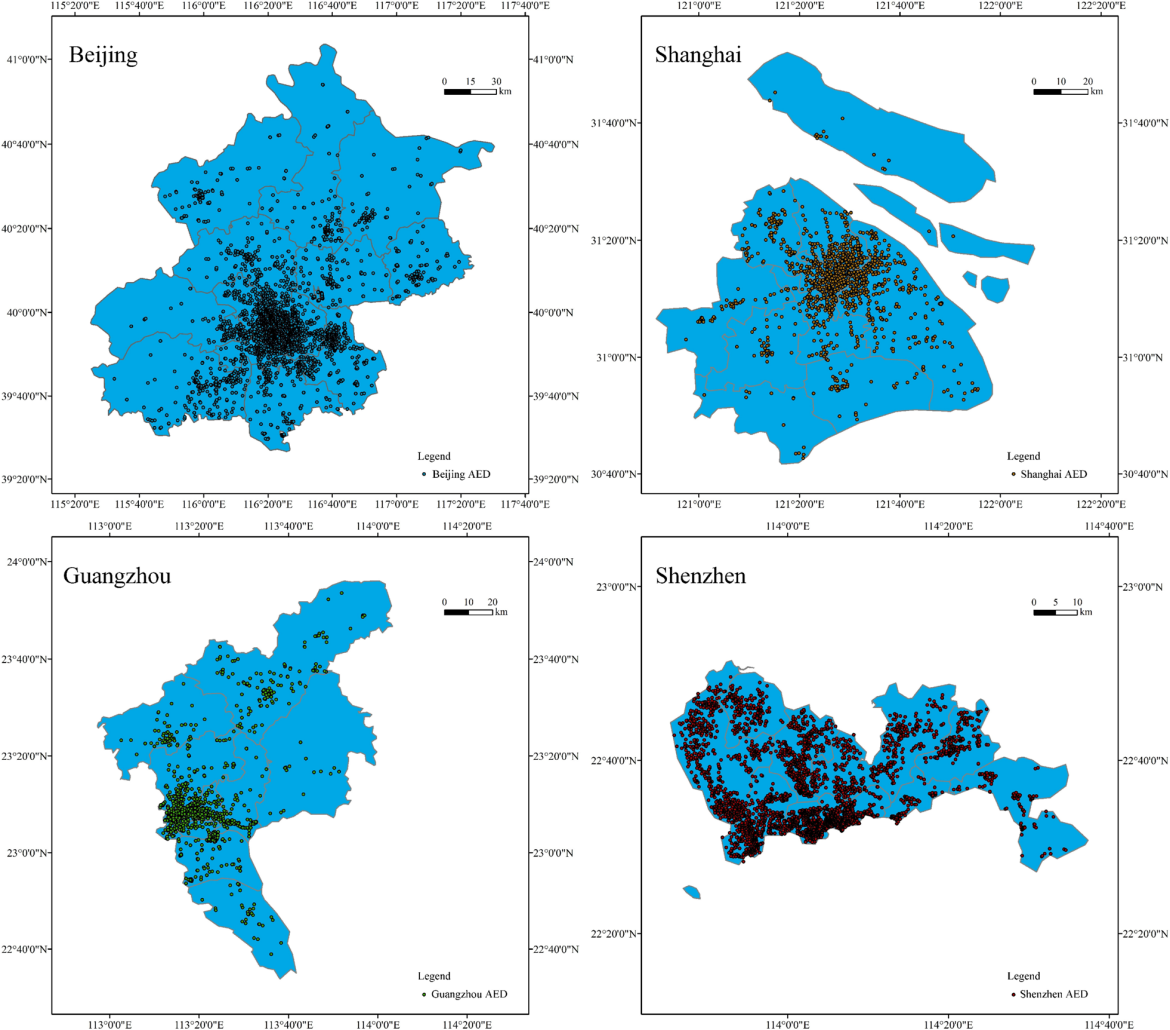
Distribution of cleaned and usable AED locations in the four cities. The map is projected in the WGS_84 coordinate system

AED location data were obtained from the WeChat mini-program “AED Map” platforms launched by city medical emergency centers. These platforms are backed by data from the respective centers. A Python script was developed in PyCharm 2023.1 to scrape AED locations in each city as of June 2024. The raw data underwent a cleaning process to obtain spatially reliable and usable AED locations. This included standardizing coordinates to a consistent geographic system, removing entries flagged as non-operational, and eliminating duplicate records based on unique identifiers and spatial proximity (detailed steps are provided in Supplementary Material S2). The cleaned data forming a city-specific AED database. Data sources are summarized in Table 3, and the spatial distribution is shown in Fig. 2.

**Table 3:** The data sources of AEDs in each city

City	Sources of AED data	Unprocessed data	Cleaned data
Beijing	Beijing 120 emergency center	5467	4415
Shanghai	Shanghai medical emergency Center & Red Cross	1251	1248
Guangzhou	Guangzhou emergency medical Command Center	1548	1225
Shenzhen	Shenzhen emergency center	7277	3944

Population data came from the WorldPop high-resolution gridded population dataset, suitable for capturing small-scale service areas of emergency facilities like AEDs. The China 2020 dataset at 100 m resolution was used [55], adjusted with data from China’s Seventh National Population Census for improved accuracy.

Road network data were downloaded from OpenStreetMap [56]. Roads within the study area and a 500 m buffer were extracted, and non-walkable roads (e.g., highways, ramps) were removed.

### Methods

Our analytical procedure follows three sequential steps to address the research questions: (1) measuring equity across four complementary dimensions, (2) aggregating these dimensions into a composite index for cross-city comparison, and (3) exploring the spatially heterogeneous drivers of the composite equity.

#### Comprehensive equity assessment model

Spatial equity is inherently a multidimensional concept, as a single indicator often fails to capture its holistic characteristics [41]. Rawls’ theory of justice provides an important philosophical foundation for spatial equity research and has been widely applied in the assessment of public resource allocation fairness [43–46]. Drawing upon Rawlsian distributive justice theory, we conceptualize AED allocation equity as an outcome-oriented form of spatial justice. Rather than mapping each indicator mechanically onto a specific Rawlsian principle, the four dimensions are interpreted as operational layers of distributive justice (Table 4), capturing complementary aspects of allocative fairness, structural inclusion, realized opportunity, and aggregate distributional balance. These dimensions collectively operationalize the multidimensional nature of spatial equity: resource allocation equity addresses whether total resources align with population needs; facility coverage equity evaluates structural service inclusion; opportunity accessibility equity measures realized access under network constraints; and spatial distribution equity examines the overall balance in the distribution of those realized opportunities. This integrated framework mitigates biases inherent in single-dimension assessments and provides a robust basis for cross-city comparisons [57, 58].

**Table 4:** Four-dimensional framework of AED allocation equity and its theoretical underpinnings

Dimension	Indicator	Rawlsian interpretive lens	Theoretical explanation
Resource allocation equity	Supply–demand ratio index	Difference principle	Focuses on the alignment between AED quantity and population size within administrative units, reflecting the right of every individual to equal basic resource guarantees
Facility coverage equity	Facility coverage index	Structural inclusion	Concerns the structural integrity of the service network, assessing whether densely populated areas fall within emergency response radii, ensuring that any spatial node can be incorporated into the service network
Opportunity accessibility equity	Accessibility index	Fair equality of opportunity principle	Measures actual access opportunities based on network impedance (walking time), ensuring that social status and spatial location do not become barriers to accessing life-saving resources
Spatial distribution equity	Gini coefficient	Distributional justice evaluation	Assesses the degree of inequality in the aggregate distribution of realized access opportunities across subdistricts, capturing structural imbalance beyond individual-level accessibility

To integrate these dimensions into a single metric, a Comprehensive Equity Index (CEI) was constructed using linear aggregation Eq. (1). The CEI is explicitly positioned as an integrated representation of AED allocation equity, rather than a causal decomposition of independent contributions. This positioning aligns with the core purpose of composite indicators—to synthesize multidimensional constructs into a transparent and interpretable summary measure for comparative analysis. Weights were determined using the entropy weight method (EWM) [59], which assigns higher importance to indicators exhibiting greater variability. Given inter-city heterogeneity, weights were calculated separately for each city. The detailed computational procedure is provided in Supplementary Material S3.

The combined use of entropy weighting and linear aggregation has been employed in prior healthcare resource equity research. For example, Hu et al. constructed a composite equity index by applying entropy-based weights to multiple indicators—such as facility supply–demand ratios, medical service coverage, and healthcare accessibility—followed by linear aggregation, demonstrating the practical feasibility of this approach in integrated equity assessment [58]. This precedent further supports the methodological appropriateness of the present design.

In composite indicator construction, correlation among indicators does not necessarily undermine methodological validity [60, 61]. According to the OECD *Handbook on Constructing Composite Indicators*, the primary principle of indicator selection is to reflect distinct aspects of the multidimensional construct under study, rather than to ensure statistical independence [62]. Moderate empirical association is therefore compatible with multidimensional measurement, provided that each indicator captures a conceptually distinct facet of the overarching construct.

The CEI was calculated as Eq. (1):

1 $${I}_{k}=S{D}_{k}\times {W}_{SD}+{C}_{k}\times {W}_{c}+{A}_{k}\times {W}_{A}+{GC}_{k}\times {W}_{GC}$$

where ${I}_{k}$ denotes the comprehensive evaluation index for subdistrict $k$; $S{D}_{k}$, ${C}_{k}$, ${A}_{k}$, and $G{C}_{k}$ represent the supply–demand ratio index, coverage index, accessibility index, and Gini coefficient for subdistrict $k$, respectively; ${W}_{SD}$, ${W}_{C}$, ${W}_{A}$, and ${W}_{GC}$ are the corresponding weights assigned to each respective indicator.

#### Calculation of equity indicators

Data were preprocessed for analysis. WorldPop population raster was converted to points, and a 200 m × 200 m grid was created to aggregate population counts. Unpopulated grids were removed.

1. Supply–demand ratio index

This measures the match between AED supply (number) and demand (population, per 10,000 people), Supply–demand ratio index was calculated as Eq. (2):

2 $$S{D}_{k}=\frac{{N}_{k}}{{P}_{k}}\times 10000$$

where ${N}_{k}$ is the number of AEDs in subdistrict $k$, ${P}_{k}$ are the total population in subdistrict $k$.

(2) Facility service coverage index

The service area for each AED was generated using network analysis based on realistic road networks. The time impedance was set to 5 min, following the “golden five minutes” principle for OHCA defibrillation, with a walking speed parameter of 100 m/min assumed for a brisk pace [63, 64]. The coverage index was then calculated by combining area coverage rate and population coverage rate, assigned weights of 20% and 80% respectively. This weighting scheme prioritizes population coverage, reflecting the common practice in public health resource accessibility assessments where serving people is often considered more critical than covering area [57, 58]. This index measures the proportion of area and population covered by AED service areas within a 5-min walk. The facility service coverage index for subdistrict $k$ is defined as Eq. (3):

3 $${C}_{k}=\sum_{i\in {U}_{k}}\left(\frac{{S}_{i}}{{S}_{k}}\times 0.2+\frac{{P}_{i}}{{P}_{k}}\times 0.8\right)$$

where ${U}_{k}$ denotes the set of grids in subdistrict $k$ that are covered by at least one AED’s 5-min service area; ${S}_{i}$ and ${p}_{i}$ are the area and population of the covered grid $i$, respectively; and ${S}_{k}$ and ${P}_{k}$ are the total area and total population of subdistrict $k$.

(3) Accessibility index

Accessibility was measured using an enhanced two-step floating catchment area (2SFCA) method, where spatial impedance refers specifically to the walking time (in minutes) from a demand grid $i$ to an AED facility $j$, calculated via network analysis. Specifically, we introduced two key modifications to the traditional 2SFCA [65]: Firstly, the supply capacity of each AED $j$ was defined by its service area extent (${S}_{j}$). Secondly, a Gaussian decay function was incorporated to replace the traditional dichotomous approach, as it more accurately reflects the continuous decay of accessibility with increasing travel time [66]. This modified model, termed the Isochrone-Gaussian 2SFCA (I-Ga2SFCA) model, calculates accessibility in two steps:

Step 1: Calculate the supply–demand ratio ${R}_{j}$ for each AED facility $j$ using Eq. (4):

4 $${R}_{j}=\frac{{S}_{j}}{\sum_{i\in \left\{{t}_{ij\le }{t}_{0}\right\}} G\left({t}_{ij},{t}_{0}\right){D}_{i}}$$

where ${S}_{j}$ denotes the service area size (supply capacity) of AED $j$, expressed in area units (km2); ${t}_{ij}$ represents the walking time from demand grid $i$ to AED $j$; ${t}_{0}$ is the time threshold (5 min), representing the maximum travel time within which an AED is considered effectively accessible; ${D}_{i}$: Population in grid $i$, as an element of the catchment area (persons); and $G\left({t}_{ij},{t}_{0}\right)$ is a Gaussian decay function that weights the interaction between grid $i$ and AED $j$ based on travel time, defined by Eq. (5):

5 $$G\left({t}_{ij},{t}_{0}\right)=\left\{\begin{array}{c}\frac{{e}^{-\left(\frac{1}{2}\right)\times {\left(\frac{{t}_{ij}}{{t}_{0}}\right)}^{2}}-{e}^{-\left(\frac{1}{2}\right)}}{1-{e }^{\left(\frac{1}{2}\right)}},\hspace{0.25em}\hspace{0.25em}\hspace{0.25em}\hspace{0.25em}{t}_{ij}\le {t}_{0}\\ 0,\hspace{0.25em}\hspace{0.25em}\hspace{0.25em}\hspace{0.25em}{t}_{ij}>{t}_{0}\end{array}\right.$$

Step 2: Compute the accessibility ${A}_{i}$ to AEDs for the population in each grid cell $i$ using Eq. (6). A higher ${A}_{i}$ value indicates better accessibility at that location.

6 $$\begin{array}{c}{A}_{i}=\sum_{j\in \left\{{t}_{ij}\le {t}_{0}\right\}} G\left({t}_{ij},{t}_{o}\right){R}_{j}\end{array}$$

The accessibility index ${A}_{k}$ for each subdistrict $k$ was then obtained by aggregating the accessibility values ${A}_{i}$ of all grid cells within it. This index represents the ease with which residents can access AED services, accounting for both supply capacity and travel impedance. This aggregation was performed using a weighted sum based on the area share and population share of each grid, as defined by Eq. (7):

7 $${A}_{k}=\sum_{i\in {U}_{k}}\left(\frac{{S}_{i}}{{S}_{k}}\times 0.2+\frac{{P}_{i}}{{P}_{k}}\times 0.8\right){A}_{i}$$

where ${U}_{k}$ denotes the set of grids in subdistrict $k$ that are covered by at least one AED’s 5-min service area;

(4) Gini coefficient

The Gini Coefficient (GC), which is widely used in economics and sociology to measure inequality in resource distribution, is used in this study to quantify the level of equal opportunity within a subdistrict, where a lower value indicates a more equal distribution [48]. It is calculated by integrating AED accessibility values and cumulative population proportions [67] using Eq. (8) and Eq. (9):

8 $$G{C}_{k}=1-{\sum}_{m=1}^{n}\left({P}_{m}-{P}_{m-1}\right)\left({D}_{m}+{D}_{m-1}\right)$$

9 $${D}_{m}=\frac{{\sum}_{i=1}^{m}{A}_{i}{r}_{i}}{{\sum}_{i=1}^{n}{A}_{i}{r}_{i}}$$

where $G{C}_{k}$ is the Gini coefficient for subdistrict $k$; $n$ is the total number of grids in $k$; $m$ is the index of grids sorted by accessibility (${A}_{i}$) in ascending order; ${r}_{i}$ is the population of grid $i$; ${D}_{m}$ is the cumulative proportion of accessibility-population product up to grid $m$ (${D}_{0}=0$, ${D}_{n}=1$); and ${D}_{m}$ is the cumulative population proportion up to grid $m$ (${P}_{0}=0$, ${P}_{n}=1$).

#### Spatial heterogeneity analysis of influencing factors based on Principal Component Analysis and Geographically Weighted Regression (PCA-GWR)

Multicollinearity among independent variables is a common challenge in multivariate regression, especially when using urban socio-economic predictors [68]. It increases the variance of parameter estimates, reduces their precision, and undermines the reliability of significance tests, making it difficult to accurately assess each variable’s effect on the outcome. Principal Component Analysis (PCA) is often used to address this issue by transforming correlated variables into a smaller set of uncorrelated components, reducing dimensionality while preserving most of the original information [69]. Additionally, traditional global regression methods like Ordinary Least Squares (OLS) assume that relationships are constant across space—an assumption often violated in spatial data. Geographically Weighted Regression (GWR) addresses this by allowing regression coefficients to vary locally, thereby capturing spatial heterogeneity and providing a more flexible modeling framework [70].

To investigate the spatially varying associations between external factors and AED allocation equity while addressing potential multicollinearity among explanatory variables, an integrated PCA-GWR approach was adopted. This methodology consists of two sequential stages:

Stage 1: Principal Component Analysis (PCA). A PCA was conducted on the set of explanatory variables to address multicollinearity by transforming them into a smaller set of uncorrelated principal components (PCs). Prior to PCA, all variables were standardized using Z-scores. The suitability of the data for PCA was statistically assessed using the Kaiser–Meyer–Olkin (KMO) measure and Bartlett’s test of sphericity. The number of retained PCs was determined based on the criterion of cumulative explained variance exceeding 80% [68]。 The component loadings, which indicate the correlation between each original variable and a PC, were examined for substantive interpretation. The detailed computational procedure for PCA is provided in Supplementary Material S4.

Stage 2: Geographically Weighted Regression (GWR). The scores of the retained PCs were then used as independent variables in a GWR model, with the CEI (${I}_{k}$) as the dependent variable. The GWR model allows regression coefficients to vary across geographic space, thereby capturing local variations in the relationships between the PCs and equity. The general form of the GWR model is expressed as Eq. (10):

10 $${I}_{k}={\beta}_{0}({u}_{k},{v}_{k})+\sum_{m=1}^{h}{\beta}_{m}({u}_{k},{v}_{k}){F}_{mk}+{\varepsilon}_{k}$$

where ${I}_{k}$ denotes the CEI of AED allocation in subdistrict $k$; $({u}_{k},{v}_{k})$ represents the spatial centroid coordinates of subdistrict $k$; ${\beta}_{0}({u}_{k},{v}_{k})$ is the spatially varying intercept; ${\beta}_{m}({u}_{k},{v}_{k})$ denotes the local regression coefficient of the $m$-th PC in subdistrict $k$; $h$ is the number of retained PCs; ${F}_{mk}$ is the score of the $m$-th PC in subdistrict $k$; and ${\varepsilon}_{k}$ represents the random error. The model employed an adaptive bi-square kernel function with an optimal bandwidth selected by minimizing the corrected Akaike Information Criterion (AICc) through a golden-section search.

## Results

### Equity model indicator items

#### Resource allocation equity

**Fig. 3:**
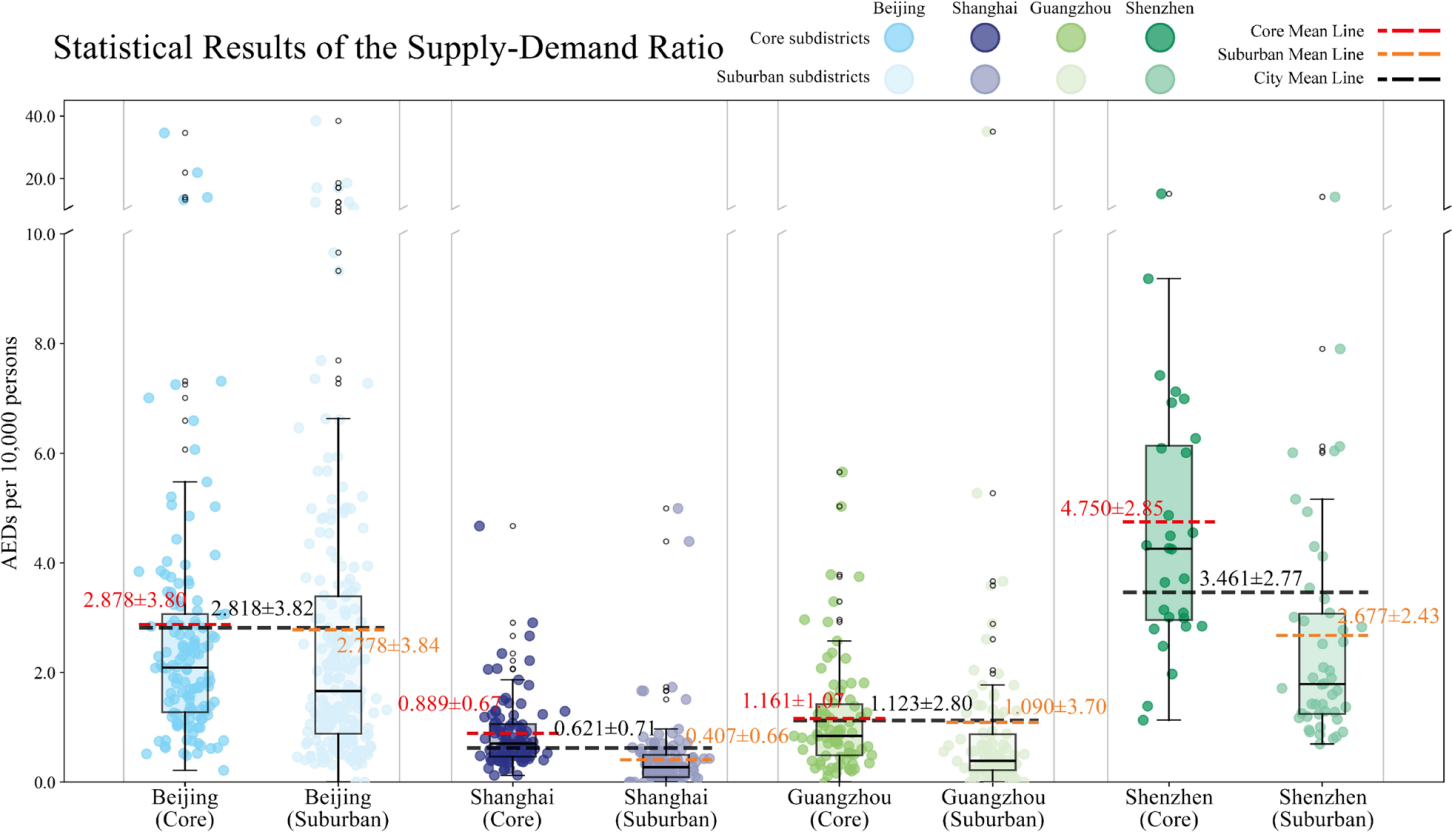
Statistical distribution of the Supply–Demand Ratio Index ($S{D}_{k}$, calculated via Eq. (2)) across core and suburban subdistricts in the four cities. The dashed horizontal line and the accompanying “Mean ± SD” label within each panel indicate the mean value and standard deviation for the respective city zone. The X-axis labels specify the city and the zone type (Core or Suburban)

The scatter plot of the Supply–Demand Ratio Index (Fig. 3) shows that all cities have extreme values deviating from the mean line, indicating significant differences in resource allocation between their internal subdistricts, which is most evident in Beijing and Shenzhen.

At the citywide level, Shenzhen achieved the highest average Supply–Demand Ratio Index, significantly outperforming the other cities, followed by Beijing. Shanghai and Guangzhou trailed considerably. Regarding core subdistricts, Shenzhen also led by a substantial margin, with Beijing ranking second, while Guangzhou and Shanghai lagged notably. Similarly, among suburban subdistricts, Beijing and Shenzhen registered comparably higher values, in contrast to the markedly lower scores in Guangzhou and Shanghai.

Variability and internal balance further distinguished the cities: Shenzhen and Shanghai showed widespread dispersion around their means across both core and suburban subdistricts, whereas Beijing and Guangzhou displayed tighter. Internally, Shenzhen and Shanghai exhibited the most pronounced core–suburban divide, with core districts substantially outperforming suburban districts. By contrast, Beijing and Guangzhou showed only marginal differences between core and suburban districts, suggesting greater intra-city equity in resource allocation.

#### Spatial coverage equity

**Fig. 4:**
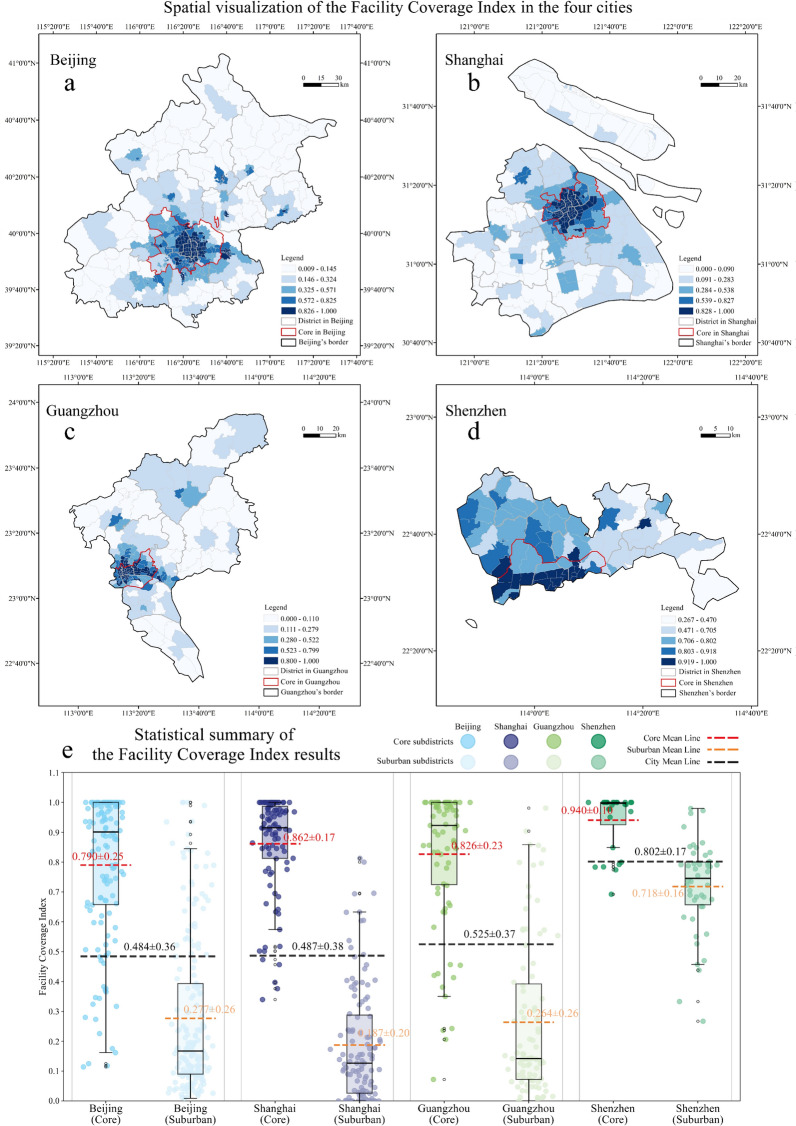
Visualization and statistical analysis of the Facility Coverage Index results (${C}_{k}$, calculated via Eq. (3)). (**a**–**d**) Spatial visualization of the Facility Coverage Index in Beijing, Shanghai, Guangzhou, and Shenzhen, respectively. The index values are classified into five levels using the Jenks natural breaks method, which optimizes data grouping by minimizing variance within classes and maximizing variance between classes, to illustrate intra-city spatial patterns. (**e**) Statistical summary of the Facility Coverage Index across core and suburban subdistricts in the four cities, facilitating both cross-city and core–suburban comparisons

The Facility Coverage Index, calculated by weighting area and population coverage rates using Eq. (3), was classified into five levels using the Jenks natural breaks method (Fig. 4a–d). The visual patterns clearly show that core districts consistently show higher coverage than suburban districts, with values declining concentrically from urban centers. Notably, certain suburban administrative centers form localized high-value clusters.

Specifically (Fig. 4e). At the citywide level, Shenzhen recorded the highest average coverage index, significantly exceeding the other three cities—approximately 1.5 times that of Guangzhou, which ranked second, and 1.6 times that of both Beijing and Shanghai. Within core subdistricts, Shenzhen continued to lead, followed by Shanghai and Guangzhou, while Beijing scored slightly lower. Similarly, in suburban subdistricts, Shenzhen maintained a clear advantage, with Beijing and Guangzhou showing moderate coverage levels and Shanghai trailing noticeably.

Variability within cities was also evident: Shenzhen demonstrated relatively balanced coverage between core and suburban subdistricts, with the smallest intra-city disparity. In contrast, Beijing, Shanghai, and Guangzhou all showed substantial gaps between core and suburban coverage, with Shanghai exhibiting the largest difference. These patterns reflect sharper spatial stratification and weaker internal equity in facility coverage outside Shenzhen.

#### Opportunity accessibility equity

**Fig. 5:**
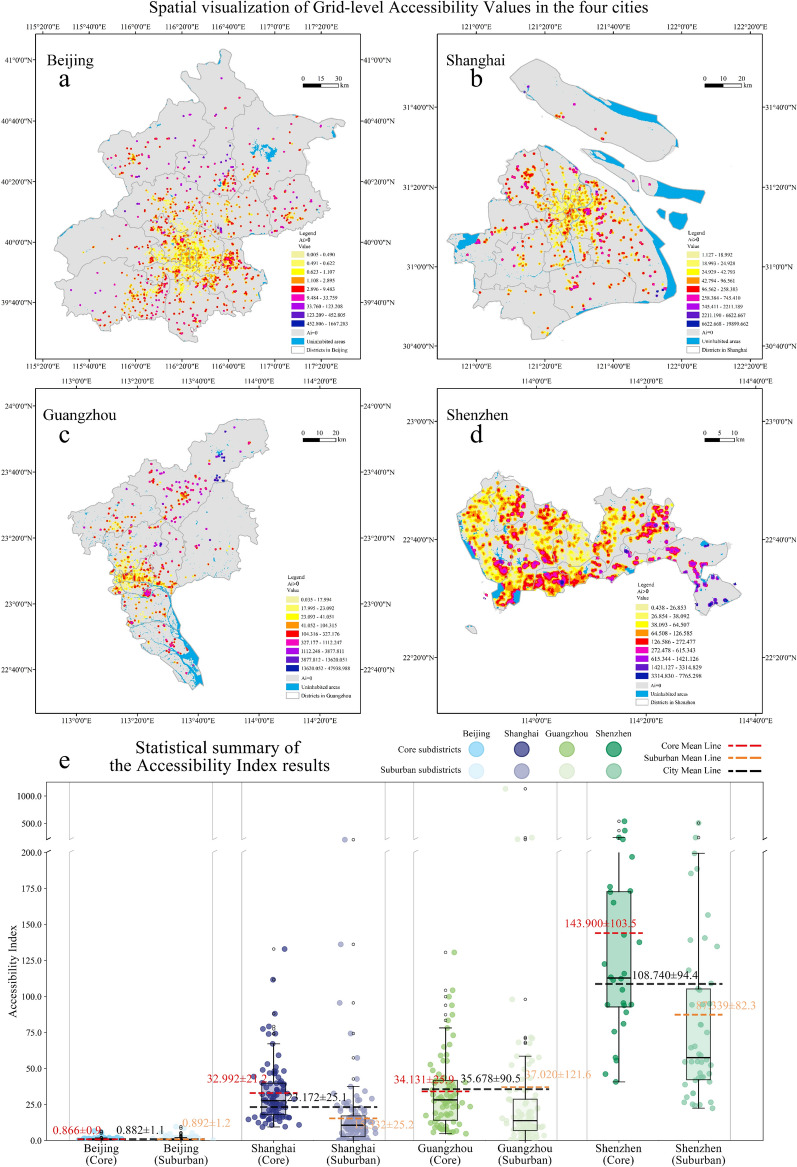
Visualization and statistical analysis of accessibility results. (**a**–**d**) Spatial visualization of grid-level accessibility values (${A}_{i}$, calculated via Eq. (6)) in Beijing, Shanghai, Guangzhou, and Shenzhen, respectively. Values are classified into nine categories using the geometric interval method, a classification scheme based on a geometric progression. This method is particularly suitable for positively skewed data, as it effectively reveals variation in the lower-value range while distinguishing the upper tail of the distribution. (**e**) Statistical summary of the subdistrict-level Accessibility Index (${A}_{k}$, calculated via Eq. (7)), facilitating cross-city and intra-city comparisons of accessibility equity

Due to the positive skew in accessibility values, geometric interval classification was applied for visualization across nine categories (Fig. 5a–d). The resulting maps reveal extensive service gaps across suburban districts of Beijing, Shanghai, and Guangzhou, where significant areas lie beyond the coverage threshold, reflecting pronounced inequity. By contrast, Shenzhen—with the exception of Dapeng New District—exhibits substantially more complete spatial coverage. Within the coverage threshold, grid-level accessibility values tend to be higher in suburban areas and lower in core urban areas, a pattern largely attributable to lower population density and reduced facility crowding in suburban zones, whereas core areas experience higher demand pressure [71].

The accessibility values were weighted by both area and population at the subdistrict level according to Eq. (7) to derive the Accessibility Index (Fig. 5e). At the citywide level, Shenzhen recorded the highest overall accessibility, substantially surpassing the other three cities. Guangzhou and Shanghai followed at moderate levels, while Beijing trailed significantly. Among core subdistricts, Shenzhen also held a strong advantage, with Guangzhou and Shanghai showing intermediate and comparable values, and Beijing registering the lowest. Similarly, within suburban subdistricts, Shenzhen remained highest, followed by Guangzhou and Shanghai, while Beijing continued to rank last.

Variability among subdistricts was pronounced in Shenzhen, Guangzhou, and Shanghai, whereas Beijing showed more uniform—though low—accessibility values. In terms of intra-urban equity, Shenzhen displayed the largest mean disparity between core and suburban subdistricts, with Shanghai also showing a considerable gap. Conversely, Guangzhou and Beijing revealed minimal core–suburban differences. Notably, in Guangzhou and Beijing, the mean accessibility index of suburban subdistricts was comparable to or even slightly higher than that of core subdistricts. This pattern may suggest that, under conditions of extremely high population density, the relative supply capacity (i.e., the service population per facility) in core areas remains under pressure despite a higher absolute number of AEDs.

#### Spatial distribution equity

**Fig. 6:**
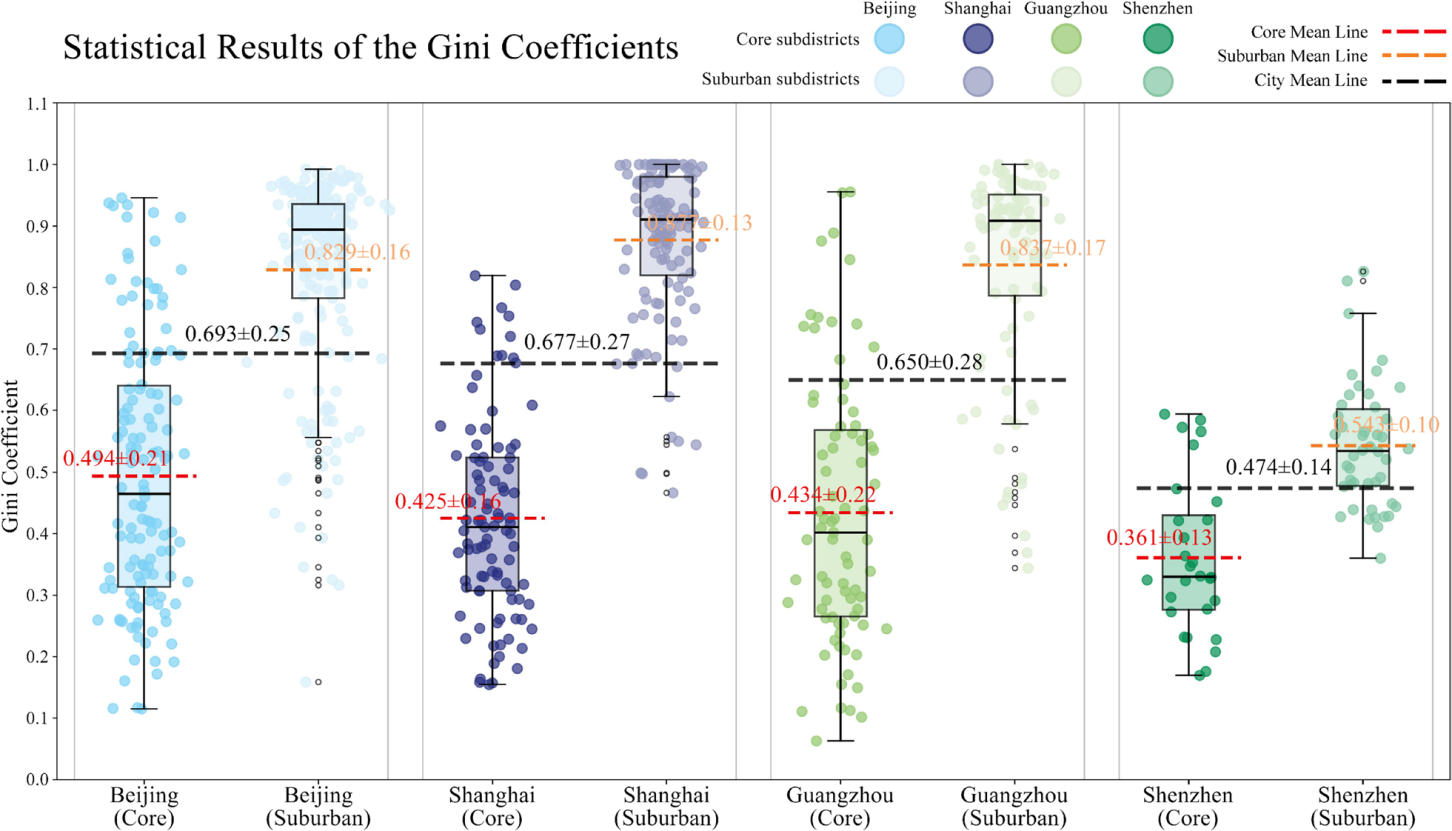
Statistical distribution of the Gini Coefficient ($G{C}_{k}$, calculated via Eq. (8)) across core and suburban subdistricts in the four cities. A higher $G{C}_{k}$ value indicates greater spatial inequality in AED accessibility within a subdistrict

According to international standards, areas with a Gini coefficient (GC) exceeding 0.4 are considered spatially inequitable [48]. The charts (Fig. 6) reveal that Shenzhen achieves the lowest mean GC among the four cities, reflecting a comparatively balanced spatial distribution. In contrast, Beijing, Shanghai, and Guangzhou all exhibit markedly higher GC values, indicating pronounced spatial inequity.

At the citywide level, Shenzhen demonstrates the most equitable distribution, while Beijing, Shanghai, and Guangzhou show substantially higher inequality. Within core subdistricts, only those in Shenzhen fall below the 0.4 threshold, suggesting relative equity; core areas in the other three cities all exceed this level. Suburban subdistricts in Beijing, Shanghai, and Guangzhou display severe inequity, with GC values surpassing 0.8. Although Shenzhen’s suburban subdistricts are less equitable than its core, they remain significantly more balanced than those in other cities.

In terms of internal variability, Shenzhen shows the smallest spread, while Beijing, Shanghai, and Guangzhou exhibit stronger variation, reflecting greater disparity among subdistricts. Shenzhen also demonstrates the narrowest core–suburban GC gap, indicating stronger internal spatial balance. Shanghai displays the largest intra-urban disparity, with Beijing and Guangzhou lying between these extremes.

### Comprehensive equity results

Having examined spatial equity across the four individual dimensions, we now integrate them into a single CEI using the EWM. This aggregated index provides a holistic measure to facilitate direct comparison of overall equity performance both across cities and between core and suburban areas within each city. Due to significant differences in AED allocation among the cities, the EWM was applied separately to each city for indicator normalization and information entropy calculation, ultimately determining the weights for each city’s indicators (Table 5).

**Table 5:** Determination of indicator weights across cities using the entropy weight method

Dimension	Indicator	City	Information entropy	Weight
Resource allocation equity	Supply–demand ration index	Beijing	0.881497	24.28%
		Shanghai	0.900697	13.76%
		Guangzhou	0.863871	14.32%
		Shenzhen	0.848267	30.24%
Spatial coverage equity	Facility coverage index	Beijing	0.870151	26.61%
		Shanghai	0.881837	16.37%
		Guangzhou	0.865131	14.19%
		Shenzhen	0.941361	11.69%
Opportunity accessibility equity	Accessibility index	Beijing	0.831240	34.58%
		Shanghai	0.590923	56.68%
		Guangzhou	0.410167	62.04%
		Shenzhen	0.782605	43.33%
Spatial distribution equity	Gini coefficient	Beijing	0.929073	14.53%
		Shanghai	0.904784	13.19%
		Guangzhou	0.910131	9.45%
		Shenzhen	0.926036	14.74%

**Fig. 7:**
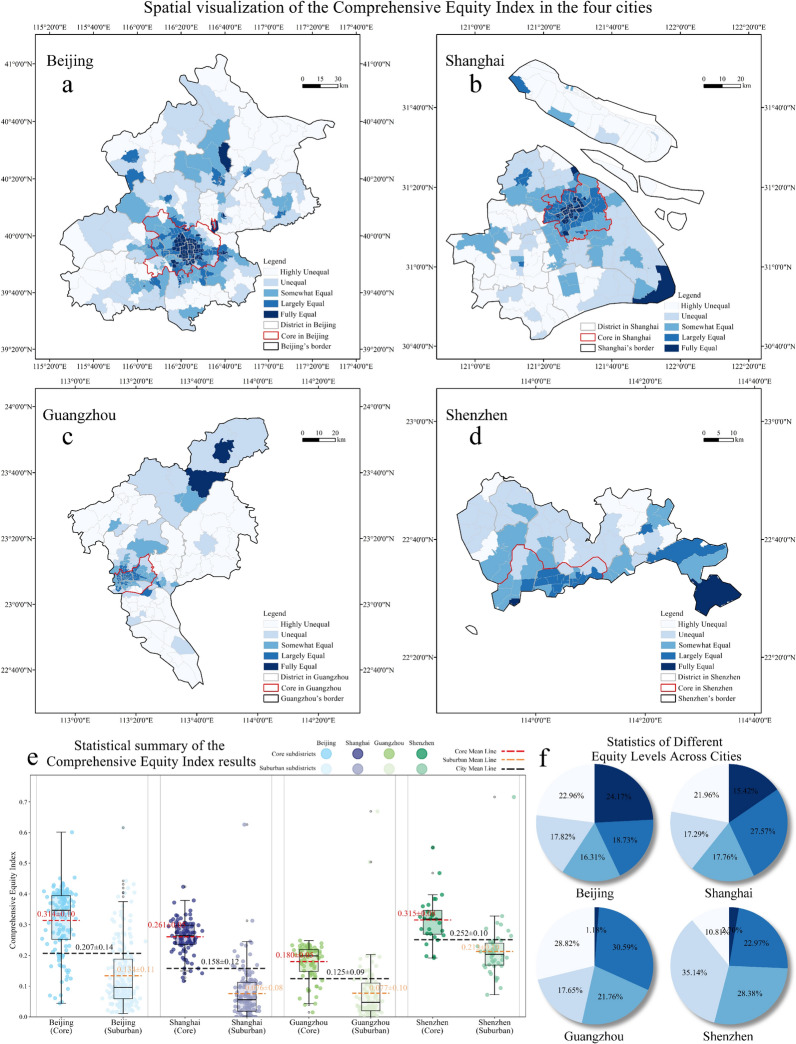
Results of the CEI (${I}_{k}$, calculated via Eq. (1)). (**a**–**d**) Spatial visualization of the index in Beijing, Shanghai, Guangzhou, and Shenzhen, respectively, classified into five levels from “Highly Unequal” to “Fully Equal” using the Jenks natural breaks method. (**e**) Statistical summary of the index across core and suburban subdistricts, facilitating cross-regional comparison. (**f**) Proportion of subdistricts at different equity levels within each city, presented as pie charts based on the classification in panels (**a**–**d**)

The indicator weights for each city were linearly weighted according to Eq. (1) to obtain the Comprehensive AED Facility Resource Allocation Equity Index for each district. Within each city, the index was classified into five levels using the Jenks natural breaks method, ranging from “Very Equitable” to “Very Inequitable”. The spatial distribution of the index, visualized in Fig. 7a–d, reveals significant heterogeneity within each city. Higher equity values are more frequently observed in core districts, while subdistricts with lower equity are more concentrated in peripheral areas. However, notable intra-regional variations exist. For instance, some core subdistricts in Beijing exhibit relatively low equity, whereas certain suburban or exurban subdistricts achieve moderate to high levels of equity.

Specifically (Fig. 7e), the overall comprehensive equity indices across all four cities remained relatively low. At the city level, Shenzhen recorded the highest overall comprehensive equity, followed by Beijing, while Shanghai and Guangzhou trailed notably. Within core subdistricts, Shenzhen and Beijing continued to lead, indicating stronger integration of AED allocation in their central areas, whereas Shanghai and Guangzhou lag behind. Similarly, among suburban subdistricts, Shenzhen maintained a clear advantage, Beijing occupied a middle position, and both Shanghai and Guangzhou performed poorly.

Based on the natural-break classification of the index (Fig. 7f), over half of the subdistricts in each city were rated as “basically equitable or above.” Specifically, Shanghai and Beijing showed similar proportions, each around 60% of their subdistricts falling into this category, while Guangzhou and Shenzhen were also comparable, both approximating 54%. The majority of these higher-rated subdistricts were located in core districts, though a limited number of suburban examples—such as southern Huairou District (Beijing), southern Dapeng New District (Shenzhen), central Conghua District (Guangzhou), and southeastern Pudong New Area (Shanghai)—also achieved this category, largely due to their lower population densities and relatively higher facility supply. Conversely, subdistricts categorized as “relatively inequitable or below” were more prevalent in Guangzhou and Shenzhen (approximately 46% and 46%, respectively), slightly higher than the proportions in Beijing (41%) and Shanghai (39%). These were predominantly concentrated in suburban districts, along with certain peripheral core subdistricts characterized by high population density and inadequate facility coverage.

### Spatial association between influencing factors and equity

To move beyond describing the equity patterns and uncover their potential drivers, we employed a PCA-GWR framework to analyze the spatially heterogeneous associations between the subdistrict-level CEI (the dependent variable) and a set of socioeconomic and urban environmental factors. The selection of these factors is detailed below.

#### Selection of explanatory variables

The spatial equity of AED allocation is influenced by multiple socioeconomic and urban environmental factors. Drawing on previous studies on inequalities in healthcare resource distribution [51, 53, 71, 72], and following the principles of representativeness and data availability, seven explanatory variables were selected for analysis (Table 6): population density, GDP per capita, GDP density, number of medical facilities (based on Point of Interest (POI) counts), number of public facilities (based on POI counts), proportion of built-up area, and road density.

**Table 6:** Selection and interpretation of influencing factors

Influencing factor	Symbol	Interpretation	Calculation formula	Data source
Population density	PD	Represents population size	Regional population / Regional area	District statistical yearbooks
GDP per capita	GDPPC	Represents residents’ economic level	Regional GDP / Regional population	District Statistical Yearbooks
GDP density	GDPD	Represents economic output per unit of land	Regional GDP / Regional area	District Statistical Yearbooks
Number of medical facilities	MF	Represents the level of medical services	Total number of facilities including clinics, hospitals, etc	Amap API [73]
Number of public venues	PV	Represents the level of public activity spaces	Total number of venues including parks, malls, schools, etc	Amap API [73]
Built-up area percentage	BAP	Represents urban development intensity	Built-up area / Regional area	Esri [74]
Road density	RD	Represents transportation conditions	Total road length / Regional area	Science Data Bank [75]

#### Principal component extraction and interpretation

Principal Component Analysis (PCA) was performed on the seven selected indicators (Table 7). Prior to PCA, the suitability of the data was confirmed: for all Z-standardized variables, the KMO values exceeded 0.6 and Bartlett’s test of sphericity was statistically significant (p < 0.05). Detailed test results are provided in Table S2 of the Supplementary Materials. For each city, three principal components (PCs) were extracted based on the criterion of cumulative proportion of variance exceeding 80%[68]. The first three PCs collectively explained over 85% of the total variance in every city, indicating that they retained the majority of the original information with limited loss.

**Table 7:** Results of PCA for the seven explanatory variables across the four cities

Variable		Beijing			Shanghai			Guangzhou			Shenzhen		
		PC1	PC2	PC3	PC1	PC2	PC3	PC1	PC2	PC3	PC1	PC2	PC3
Eigenvalue		3.894	1.657	0.726	3.346	1.804	1.067	3.021	1.888	1.111	3.380	2.049	0.934
Proportion of variance (%)		55.46%	23.60%	10.34%	47.57%	25.65%	15.17%	42.90%	26.81%	15.77%	47.63%	28.88%	13.17%
Cumulative proportion of Variance (%)		55.46%	79.06%	89.40%	47.57%	73.22%	88.39%	42.90%	69.71%	85.48%	47.63%	76.51%	89.68%
Principal component loadings	PD	0.857	-0.039	-0.435	0.749	-0.166	-0.571	0.799	0.233	-0.389	0.800	-0.018	-0.460
	GDPPC	0.630	-0.450	0.601	0.624	-0.200	0.728	0.333	-0.396	0.828	0.512	-0.522	0.660
	GDPD	0.820	-0.432	0.129	0.868	-0.313	0.272	0.831	-0.055	-0.364	0.744	-0.505	0.255
	MF	0.591	0.739	0.080	0.441	0.811	0.140	-0.215	0.877	0.322	0.430	0.837	0.256
	PV	0.392	0.828	0.305	0.093	0.945	0.096	-0.417	0.831	0.245	0.263	0.901	0.290
	BAP	0.879	0.075	-0.228	0.805	0.259	-0.315	0.796	0.386	-0.068	0.914	0.087	-0.175
	RD	0.902	-0.173	-0.086	0.890	-0.149	-0.090	0.854	0.257	-0.190	0.915	-0.021	-0.203

To further evaluate the robustness of the PCA-GWR results, we assessed the statistical significance of the local coefficient estimates. A detailed summary of the significance levels (percentage of subdistricts with p < 0.05) for each principal component across the four cities is provided in Supplementary Material S6 (Table S5)

The principal component loadings of the three retained PCs for the four cities are shown in Table 7. Examination of the loadings reveals consistent patterns across cities. The first principal component (PC1) is primarily dominated by RD, BAP, PD, and GDPD. This component was therefore interpreted as the Degree of Urbanization Component (DU), reflecting the overall intensity of urban development as well as the concentration of population and economic activities per unit area. The second principal component (PC2) is mainly characterized by high loadings of MF and PV. Accordingly, this component was defined as the Public Service Facility Provision Component (PSFP), representing the level of public and healthcare service supply. The third principal component (PC3) exhibits strong positive loadings on GDPPC and negative loadings on PD. This component was interpreted as the Wealth–Population Density Trade-off Component (WPDT), capturing the contrast between economic affluence and population concentration across subdistricts. Through PCA, the original seven correlated indicators were thus transformed into three mutually orthogonal principal components, which were subsequently used as explanatory variables in the PCA-GWR analysis.

#### Spatially varying effects of principal components based on PCA-GWR

**Fig. 8:**
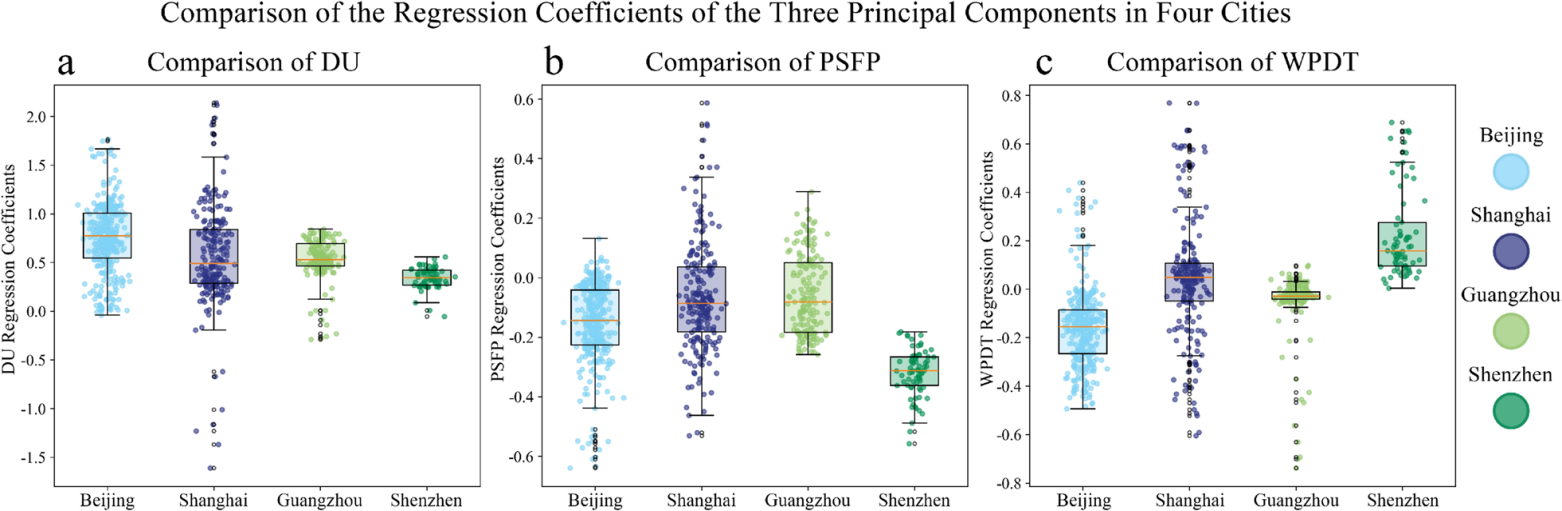
Comparison of the regression coefficients of the three principal components in four cities

**Fig. 9:**
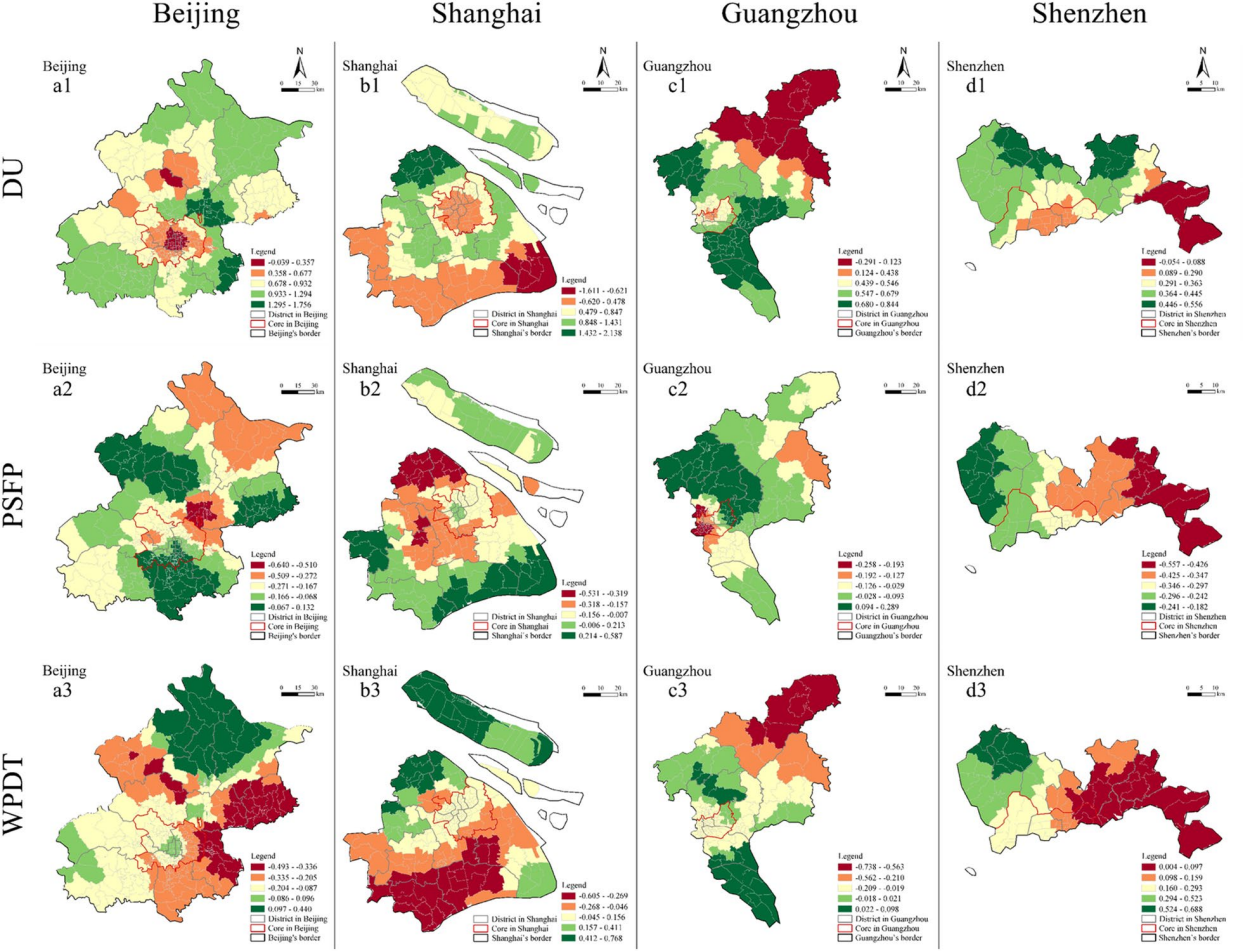
Spatially varying local regression coefficients from the PCA-GWR model. The maps depict the spatial distribution of local coefficients for (**a1**-**d1**) the DU component, (**a2**-**d2**) the PSFP component, and (**a3**-**d3**) the WPDT component across subdistricts in Beijing (**a1**-**a3**), Shanghai (**b1**-**b3**), Guangzhou (**c1**-**c3**), and Shenzhen (**d1**-**d3**). Coefficient values are classified into five categories using the Jenks natural breaks method

The PCA-GWR model fitting results for each city, using the three extracted principal components as explanatory variables, are summarized in Table 8. Compared with the conventional PCA-OLS model, the PCA-GWR model exhibits improved goodness-of-fit, indicating its advantage in accounting for multicollinearity among explanatory variables and capturing spatial heterogeneity. Accordingly, subsequent analyses were based on the PCA-GWR results. A comparison of regression coefficients across cities is shown in Fig. 8, while the spatial distributions of local regression coefficients for each principal component are illustrated in Fig. 9.

1. Degree of urbanization component (DU)

**Table 8:** Comparison of model performance between PCA-OLS and PCA-GWR for the four cities

Performance metric	Beijing		Shanghai		Guangzhou		Shenzhen	
	PCA-OLS	PCA-GWR	PCA-OLS	PCA-GWR	PCA-OLS	PCA-GWR	PCA-OLS	PCA-GWR
Residual Sum of Squares (RSS)	96.445	58.692	73.325	34.337	110.337	72.371	53.1	36.356
Log-likelihood	-265.583	-183.385	-189.047	-107.868	-204.478	-168.63	-92.721	-78.705
AICc	541.351	477.337	388.383	316.813	419.321	380.874	196.325	185.598
R^2^	0.709	0.823	0.657	0.84	0.351	0.574	0.282	0.509
Adjusted R^2^	0.706	0.794	0.652	0.803	0.339	0.523	0.252	0.425

From a cross-city comparison perspective (Fig. 8a), the PCA-GWR results for the DU reveal a predominant positive association with comprehensive AED equity across the four cities, indicating that higher urban development intensity generally correlates with greater equity. However, significant spatial heterogeneity exists within each city.

From an intra-city spatial pattern perspective (Fig. 9a1-d1), in Beijing, stronger positive associations are observed in northern and southern outer suburbs, as well as parts of the eastern inner suburbs. Shanghai exhibits pronounced positive associations in northern Jiading and Baoshan Districts, and in several inner suburban areas surrounding the urban core, while parts of eastern Pudong New Area show localized negative associations. In Guangzhou, relatively high positive coefficients are mainly distributed in the city’s southern regions and inner suburban areas north of the urban core. Shenzhen’s higher regression coefficients are primarily found in its northern and western parts. A common pattern across all cities is that regression coefficients within urban core areas are consistently moderate to low. This suggests that the association between urban development intensity and comprehensive AED equity is relatively limited in highly developed city centers compared to surrounding suburban regions.

(2) Public service facility provision component (PSFP)

The PCA-GWR results for the PSFP reveal marked spatial heterogeneity in its association with comprehensive AED equity across and within cities. At the inter-city level (Fig. 8b), Beijing and Shenzhen exhibit predominantly negative associations, while Shanghai and Guangzhou show mixed patterns of both positive and negative coefficients.

Spatial patterns within each city are distinct (Fig. 9a2-d2). In Beijing, pronounced negative associations are concentrated in Shunyi District, northern Miyun and Huairou, and the Shijingshan–Haidian border area, whereas Yanqing, Daxing, and southern Pinggu display near-zero or weakly positive associations. Shanghai shows strong spatial variation: negative associations cluster in northern Jiading and Baoshan Districts and the junction of Qingpu, Songjiang, and Xuhui, while positive associations appear in southeastern Shanghai and southern Qingpu. In Guangzhou, negative associations are mainly found in the western urban core and northern Zengcheng, contrasting with positive associations in northern Huangpu, Baiyun, and Huadu. Shenzhen presents a consistent negative association across the entire city, with the strength of association gradually decreasing from east to west, indicating a clear spatial gradient.

(3) Wealth–population density trade-off component (WPDT)

The PCA-GWR results for the WPDT show substantial variation across cities (Fig. 8c). Beijing exhibits predominantly negative associations, Shanghai shows a mixed pattern of both positive and negative coefficients, Guangzhou displays coefficients clustered around zero indicating a weak association, and Shenzhen demonstrates consistently positive associations across all subdistricts.

At the intra-city level, distinct spatial patterns are observed (Fig. 9a3-d3). In Beijing, negative associations are concentrated in eastern areas and the junction of Yanqing and Changping Districts, while positive associations appear in northern regions. Shanghai shows more pronounced heterogeneity, with positive associations in the northern parts and eastern Pudong, and negative associations in southern regions and the junction of Jiading, Baoshan, and Putuo Districts. In Guangzhou, negative associations are primarily located in northern outer suburbs, while weak positive associations occur in southern outer suburbs and central Baiyun District. Shenzhen maintains consistently positive associations citywide, with a clear west-to-east gradient of decreasing coefficient magnitude.

## Conclusions

### Significant multidimensional disparities with shenzhen outperforming others

Shenzhen significantly outperformed the other three cities across all four equity dimensions—resource allocation, spatial coverage, opportunity accessibility, and spatial distribution—indicating that its high investment and refined management have effectively balanced both total quantity and equitable distribution. In contrast, Beijing, Shanghai, and Guangzhou showed notable internal disparities. Beijing exhibited prominent differences between core and suburban districts in spatial coverage and spatial distribution. Shanghai demonstrated a pattern of “high in core districts, low in suburban districts” in resource allocation, spatial coverage, and spatial distribution. Guangzhou showed evident gradients in spatial coverage, opportunity accessibility, and spatial distribution. This finding underscores that facility equity is shaped by multiple dimensions; advantages in one dimension cannot compensate for deficiencies in others. Enhancing equity, therefore, requires a transition to a refined strategy emphasizing “multi-dimensional coordination and weakness prioritization”.

### Overall comprehensive equity remains low with pronounced core–periphery disparities

The comprehensive spatial equity of AED allocation in China’s first-tier cities remains generally low. Among the four cities, Shenzhen demonstrated the highest level of equity, significantly outperforming the others. Beijing ranked second, while Shanghai and Guangzhou lagged considerably. More importantly, all four cities exhibited a distinct “core–periphery” gradient, wherein the core districts showed significantly higher comprehensive equity than suburban districts. This equity gap was particularly acute in Beijing and Shanghai. These findings reveal that despite substantial investments under China’s rapidly advancing PAD program, the equitable distribution of resources in first-tier cities remains a serious challenge. The growing disparities in intraregional development are increasingly perceived as a critical constraint affecting the equitable distribution of prehospital emergency care resources.

### Spatially heterogeneous effects of urban development, public services, and socioeconomic structure on AED equity

Based on the PCA-GWR analysis, the comprehensive equity of AED allocation exhibits pronounced spatial heterogeneity and is shaped by multiple urban structural dimensions. Firstly, degree of urbanization shows a generally positive association with comprehensive AED equity, while its association weakens in highly developed urban core areas, indicating diminishing effectiveness in central districts. Secondly, public service facility provision displays inconsistent associations with AED equity, with predominantly negative or spatially heterogeneous patterns across cities, suggesting that higher facility provision does not necessarily correspond to greater equity. Thirdly, the wealth–population density trade-off component demonstrates marked city-specific differences, ranging from predominantly negative or weak associations to consistently positive associations, underscoring the non-uniform role of socioeconomic structure in shaping AED equity. These results collectively highlight the necessity for spatially differentiated policy interventions within China’s PAD program.

## Discussions

### Mechanisms of spatially heterogeneous influences on comprehensive AED equity

Across the four megacities, the DU component shows a significant positive association with comprehensive AED equity at the macro scale, indicating that areas with higher development intensity generally benefit from more equitable AED access. Concentrated populations, economic activity, and well-established built environments appear to be focal points for current AED deployment, a finding consistent with prior studies reporting positive correlations between high population density/urban form metrics and the equity of medical facilities [51, 53]. However, the PCA-GWR model reveals a critical spatial heterogeneity: the strength of the association between DU and equity is consistently weaker within urban core districts. Despite hosting the highest concentrations of population and economic activity, further intensification in these core areas does not yield proportional gains in equity. This spatial pattern conceptually resonates with discussions in urban systems and infrastructure allocation literature. It aligns with the broader theory of ‘sub-linear scaling’ in cities, where the per-capita need for certain types of infrastructure grows slower than population size due to agglomeration efficiencies [76]. In our context, the weakened DU-equity association in cores suggests that the marginal return on equity from further increases in general urban development intensity may be diminishing. For instance, Ning et al.’s study on the evolution of Chinese urban systems found that since 2003, healthcare facilities have exhibited a “sub-linear” scaling relationship with city size in some contexts—though not universally—partly due to governmental safety-net provisions for essential infrastructure [77]. Similarly, the stronger DU effects observed in certain suburban districts in our study may reflect compensatory AED allocation policies aimed at mitigating core-periphery spatial disparities.

The association between PSFP and comprehensive AED equity varies substantially both across and within cities. Predominantly negative correlations in Beijing and Shenzhen, together with pronounced spatial heterogeneity in Shanghai and Guangzhou, indicate a frequent misalignment between general public service infrastructure and equitable AED access. In areas with negative PSFP associations, public venues and medical facilities may not have been systematically equipped with AEDs despite their high service intensity, suggesting gaps between policy intent and on-the-ground implementation. By contrast, positively associated zones likely reflect more effective integration of AEDs within existing public service environments, particularly in high-activity public spaces. Prior empirical evidence indicates that public venues play a critical role in AED deployment and use, with AEDs involved in OHCA events most frequently retrieved from such locations [24, 78]. From this perspective, areas exhibiting negative PSFP–equity relationships—such as large portions of Beijing and Shenzhen—may require more targeted AED installation within public facilities to bridge this gap.

The relationship between the WPDT and comprehensive AED equity exhibits the most pronounced inter-city variation among the three components. Predominantly negative associations observed across much of Beijing suggest that, in areas with relatively lower economic wealth, equitable AED outcomes may be strongly supported by policy-driven minimum provision strategies. This implies that governmental allocation mechanisms can play a critical role in sustaining baseline equity levels, even where local economic capacity is limited. By contrast, the consistently positive associations identified across Shenzhen point to a different pathway, in which higher regional affluence is closely linked to improved AED equity. This pattern likely reflects socio-economic advantages commonly present in wealthier areas, including stronger public awareness of emergency preparedness and a greater willingness to invest in health-related resources such as AEDs. Similar tendencies have been documented in previous studies, which report more equitable AED distribution in economically advantaged neighborhoods [17, 79].

### Policy differentiation and its association with equity: a comparative analysis

China’s current PAD program remains government-led, with facility allocation heavily reliant on the design and implementation of relevant policies. Although quantitatively assessing policy impact involves methodological challenges, a comparative analysis of implementation timelines and policy content (Table 9) offers valuable insights.

**Table 9:** Summary of AED allocation policies in various cities

City	Year	Document title	Key content
Beijing	2017	Regulations on pre-hospital medical emergency services in Beijing	Mandates AED placement in specified public venues (e.g., transport hubs, schools, scenic spots)
	2020	Implementation plan for strengthening the pre-hospital medical emergency service system in Beijing	Promotes the configuration of AEDs in public places
	2021	Three-year action plan for building social emergency response capacity in key public places in Beijing (2021–2023)	Aims for > 5,000 AEDs citywide (> 20 units/100,000 people). Requires coverage in key venues and encourages wider placement
Shanghai	2016	Shanghai regulations on emergency medical services	Mandates first aid equipment (incl. AEDs) in densely populated public places and large enterprises
	2025	Key tasks for shanghai medical emergency work in 2025	Aims to add 7,500 AEDs, achieving a target of > 50 units/100,000 people
	2025	Key tasks for shanghai health work in 2025	Reiterates the goal of deploying 7,500 additional AEDs across the city
Guangzhou	2023	Guangzhou regulations on social emergency medical management	Mandates AEDs in government service halls, transport hubs, large malls, schools, etc. Encourages broader installation and donations
	2024	Guangzhou specifications for the configuration of Automated External Defibrillators (AED) in public places (Interim)	Specifies detailed AED configuration standards (quantity, location, management) for different types of public venues
Shenzhen	2017	Public place AED configuration project	First city-wide systematic AED deployment program in China
	2023	Construction and management specifications for automated external defibrillators in public places (DB4403/T 318–2023)	Specifies mandatory AED service radii (≤ 300 m for Category 1 venues, ≤ 600 m for Category 2) and configuration density
	2023	Shenzhen special economic zone regulations on medical emergency response	Requires government planning for AED placement in public transport venues. Encourages installation in densely populated areas

Firstly, Shenzhen’s comprehensive and detailed configuration policies have driven its superior equity outcomes. As the first city to systematically implement a public defibrillation program (2017), Shenzhen introduced the “Public AED Configuration Project," mandating AED deployment in high-traffic venues such as scenic areas and schools. In 2023, the city further issued local standards specifying service radii and configuration density, shifting from extensive placement to optimized coverage. This policy framework forms a closed loop of “systematic standards—sustained investment—dynamic adjustment,” contributing to Shenzhen’s high equity performance. By October 2024, AEDs in Shenzhen had cumulatively aided in the successful resuscitation of 115 OHCA patients, making it the most successful implementation model under China’s PAD program [80]. Secondly, Beijing’s policy closely followed Shenzhen’s, resulting in the second-highest equity. Although Beijing introduced similar policies in 2017, with additional documents in 2020 and 2021 accelerating facility deployment, its extensive administrative area has hindered the transition to fully optimized allocation, leaving equity at a secondary level [53, 81]. Thirdly, Shanghai and Guangzhou exhibited lower equity, likely influenced by delayed policy implementation. Shanghai initially endorsed AED configuration in 2016 but did not substantively advance deployment until the 2025 “*Work Priorities*” directives, resulting in nearly a decade of policy stagnation and a lower equity index. Guangzhou only mandated AED configuration in public venues through legislation in 2023, with detailed management guidelines issued in 2024. This delayed response likely contributed to its lowest equity ranking among the four cities.

It should be noted that this study did not quantify policy variables—such as fiscal input intensity or enforcement frequency—in relation to equity. Interpretations regarding potential policy influence are drawn primarily from policy text and implementation timelines. Future research could develop quantitative policy effectiveness indicators to precisely elucidate policy-driven mechanisms. 

### Prospects

#### Equity considerations for vulnerable groups

Vulnerable groups are frequently overlooked in public facility allocation. To advance equity-oriented research and practice, future studies could strengthen the focus on AED accessibility for these populations through the following approaches: Firstly, regarding equity for individuals with a history of heart disease, who face a higher risk of OHCA [82, 83], developing geographic distribution models of high-risk cardiac populations is crucial. This could be achieved by utilizing anonymized community/subdistrict-level health records (e.g., chronic disease registries) combined with field health surveys. Such models would provide a foundation for assessing the alignment of AED deployment with the spatial distribution of this vulnerable group. Secondly, concerning equity for the elderly, who face elevated OHCA risk [84], integrating multi-source data—such as community surveys and medical records—through machine learning to establish age-stratified distribution models could inform a risk-weighted equity evaluation framework. Thirdly, for equity regarding people with disabilities, establishing geospatial databases of disabled populations through civil administration registries and community surveys would enable the development of accessibility-focused service evaluation systems. Additionally, exploring incentive mechanisms (e.g., recognition for assisting in defibrillation) may be considered to improve rescue efficiency for this group.

#### Equity under dynamic demand conditions

Addressing dynamic demand is a critical consideration for enhancing the effectiveness of AED deployment. Future studies could incorporate population mobility into AED equity research through the following avenues: Firstly, innovating deployment modes by installing AEDs on mobile carriers such as public transit vehicles presents a promising strategy. Spatial coupling analysis that integrates vehicle trajectories and human flow patterns could evaluate the efficacy of mobile AEDs in meeting transient and dynamic demand. Secondly, leveraging real-time data—such as population heat maps and mobile phone signaling—to construct spatiotemporal models of crowd movement can quantify the service decay of static AEDs during peak hours, holidays, and special events. This approach allows for evaluating the equity implications of temporal population shifts and identifying high-flow areas where smart AED placement might be beneficial.

#### International comparison and urban scaling perspectives

**Fig. 10:**
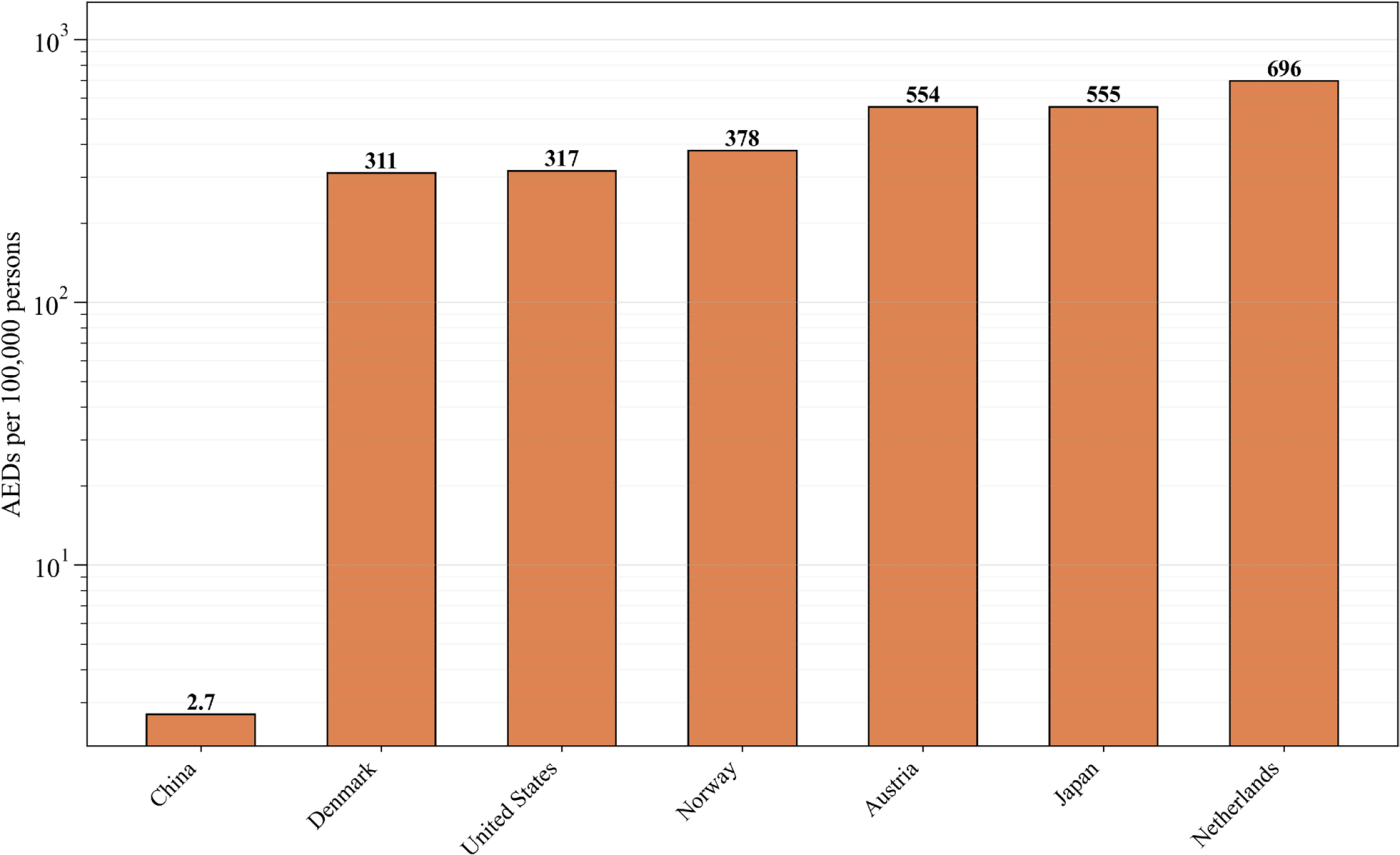
Comparative AED density (units per 100,000 people) in China and selected countries. Source:[85]

Firstly, placing China’s PAD progress in a global context reveals a significant deployment gap. Current estimates suggest China has only about 2.7 AEDs per 100,000 people (Fig. 10), far below the densities in countries with mature PAD programs such as Japan (555 per 100,000) or the Netherlands (696) [85]. This gap underscores the urgent need to accelerate the national PAD program and improve the AED deployment system.

Secondly, our findings invite consideration of urban scaling principles for future resource planning. We observed that the positive association between Degree of Urbanization (DU) and comprehensive equity was weaker in city cores than in suburban areas. This spatial heterogeneity suggests that the relationship between urban intensity (e.g., population density) and equitable AED comprehensive equity may not be linear. It aligns with the concept of sublinear scaling, where per-capita infrastructure needs may increase at a slower rate than population growth in denser areas due to efficiencies of agglomeration and targeted equity policies [76]. However, rigorously testing AED-specific scaling laws in China requires overcoming a major data barrier: the lack of a unified national AED registry. Establishing such a national database is therefore crucial for enabling future international and cross-city comparisons and for advancing urban scaling research on AED provision in China.

#### Towards outcome-oriented equity: integrating OHCA risk and environmental determinants

The ultimate objective of equitable AED allocation is to improve survival outcomes from OHCA. Future research could therefore strive to shift from evaluating input or opportunity equity towards outcome equity, directly linking AED deployment to survival rates. A critical step is incorporating fine-grained, spatially explicit OHCA incidence data. International studies reveal stark contrasts in coverage relative to need; for instance, approximately 40% of OHCAs in Milan occurred within AED service areas, compared to only 7.5% in Taoyuan [18, 23]. Such disparities underscore the necessity of grounding equity assessments in actual OHCA geography. Furthermore, the spatial distribution of OHCA is influenced by environmental risk modifiers. Substantial evidence identifies extreme temperatures and air pollutants (e.g., PM₂.₅, NO₂) as significant predictors of OHCA onset [86, 87]. Integrating high-resolution spatial layers of these environmental risk factors (e.g., global air quality data from NASA SEDAC, or regional climate datasets from China’s National Tibetan Plateau Data Center) could enable a more nuanced, risk-stratified equity assessment. This approach would move beyond using population density as a sole proxy for demand, towards a smarter placement strategy that prioritizes areas with higher predicted OHCA risk due to environmental stressors. This would align resource allocation more closely with probable need and potential life-saving impact.

#### Conclusions for policy

Based on the findings of significant core-periphery disparities and the spatially heterogeneous effects of Degree of Urbanization, public service provision, and socioeconomic structure on comprehensive equity as revealed by the PCA-GWR analysis, the following implications can be drawn for improving AED spatial allocation equity within China’s PAD program: Firstly, the development of differentiated configuration standards for core and suburban districts appears warranted. While higher density in core areas may reflect population concentration, suburban districts must be guaranteed a mandatory minimum AED density to address geographic inequity. These standards could be dynamically adjusted based on local demographic structure (e.g., aging index) and geographic conditions. Secondly, to improve coverage in geographically isolated suburban areas, deploying networked AED distribution points and exploring innovative delivery methods (e.g., drones) represent potential pathways to enhance equity in hard-to-reach areas. Concurrently, integrating AED deployment into new suburban transport hubs, and community centers can leverage urbanization to improve equity. Thirdly, implementing a mechanism that links AED configuration to land development could be considered. This might involve requiring real-estate developers and large project planners to finance, install, and maintain AEDs in proportion to projected pedestrian volume and construction scale, formalizing this as a precondition for planning approval.

### Limitations

This study systematically evaluates AED allocation equity across four major Chinese first-tier cities with relatively mature PAD systems. While these cities serve as important benchmarks, the findings should be interpreted with caution when generalized to cities at different developmental stages or under differing institutional contexts. The following limitations are acknowledged:

Firstly, due to data constraints, equity is evaluated based on facility provision and access opportunity rather than health outcomes. The lack of precise OHCA incidence data precludes an assessment of outcome-based equity—i.e., how well AED deployment aligns with actual survival rates. Secondly, the 5-min service radius and related assumptions do not fully capture real-world contextual barriers such as building layouts, traffic conditions, or user capability differences, which may affect actual accessibility. Thirdly, the AED location data, drawn from official city platforms at a single point in time, may not be fully comprehensive; potential under-coverage could influence metrics such as the supply–demand ratio and coverage index.

Finally, it should be noted that the four equity indicators in this study are empirically related in their derivation, yet they represent analytically distinguishable levels of spatial justice. This multidimensional integration approach aligns with the research tradition of public facility equity assessment, in which composite indices are commonly employed to capture the holistic nature of distributive justice [58]. To ensure the robustness of the composite index, systematic checks were performed: variance inflation factors for all indicators were below 7.5, indicating that multicollinearity remained within an acceptable range; and a PCA-derived composite index showed high correlation (Spearman’s ρ > 0.9) with our entropy-weighted index (see Supplementary Material S5), confirming that the core findings are insensitive to the choice of aggregation method. Future research could explore more sophisticated aggregation approaches, such as structural equation modeling or multiple indicators multiple causes models, to more precisely disentangle the independent effects of each dimension and further advance the understanding of equity mechanisms in AED allocation.

## Supplementary Information

Additional file1.

## Data Availability

The datasets used in this study include publicly available data and restricted data available upon request. The gridded population data are available from the WorldPop project (https://hub.worldpop.org/geodata/summary?id=24926); road network data from OpenStreetMap (https://www.openstreetmap.org); Points of Interest for medical and public facilities from the Amap API (https://lbs.amap.com); built-up area percentage from the Esri Land Cover Explorer (https://livingatlas.arcgis.com/landcoverexplorer); and road density data from the Science Data Bank (10.57760/sciencedb.02938). The location-based AED data were obtained from third-party WeChat mini-programs and, due to provider terms of service and privacy restrictions, cannot be shared publicly but are available from the corresponding author upon reasonable request.

## Abbreviations

OHCA: Out-of-hospital cardiac arrest
AED: Automated external defibrillator
CPR: Cardiopulmonary resuscitation
EMS: Emergency medical services
PAD: Public access defibrillation
GIS: Geographic information system(s)
2SFCA: Two-step floating catchment area
I-Ga2SFCA: Isochrone-gaussian two-step floating catchment area
GC: Gini coefficient
POI: Point of interest
PCA: Principal component analysis
EWM: Entropy weight method
GWR: Geographically weighted regression
