# Supplementary material for: Spatial equity in the allocation of lifesaving resources: a cross-sectional spatial evaluation of automated external defibrillators in four first-tier Chinese cities
Source: Int J Health Geogr. 2026 May 2;25:34. doi: 10.1186/s12942-026-00470-w (PMC13267634; doi:10.1186/s12942-026-00470-w)
Supplement: Supplementary file 1 — Additional file1. [file 12942_2026_470_MOESM1_ESM.docx]

**Supplementary Material for:**

**Spatial Equity in the Allocation of Lifesaving Resources: A Cross-Sectional Spatial Evaluation of Automated External Defibrillators in Four First-Tier Chinese Cities**

**Aiping Gou^1^, Lei Wang^1^,** **Jiangbo Wang^2*^, Chunyan Gou^3*^, Jing Li^4^**

* Correspondence:

Jiangbo Wang

jumbo@njtech.edu.cn

Chunyan Gou

335719554@qq.com

^1^ College of Ecological Technology and Engineering, Shanghai Institute of Technology, Shanghai 201418, China

^2^ College of Architecture, Nanjing Tech University, Nanjing 211816, China

^3^ Chongqing Traditional Chinese Medicine Hospital, Chongqing 400021, China

^4^ Department of Acupuncture, Yueyang Hospital of Integrated Traditional Chinese and Western Medicine, Shanghai University of Traditional Chinese Medicine, Shanghai 200437, China

**Table of Contents**

**S1. High-resolution Maps of the Study Areas with District Labels**

**S2. Detailed Data Processing and Cleaning Steps for AED Locations**

**S3. Methodological Details of the Entropy Weight Method**

**S4. Methodological Details of the PCA-GWR Framework**

**S5. Diagnostics and Robustness Checks for the Comprehensive Equity Index (CEI)**

**S6. Summary of Local Coefficient Significance for PCA-GWR Models**

**S1. High-resolution Maps of the Study Areas with District Labels**


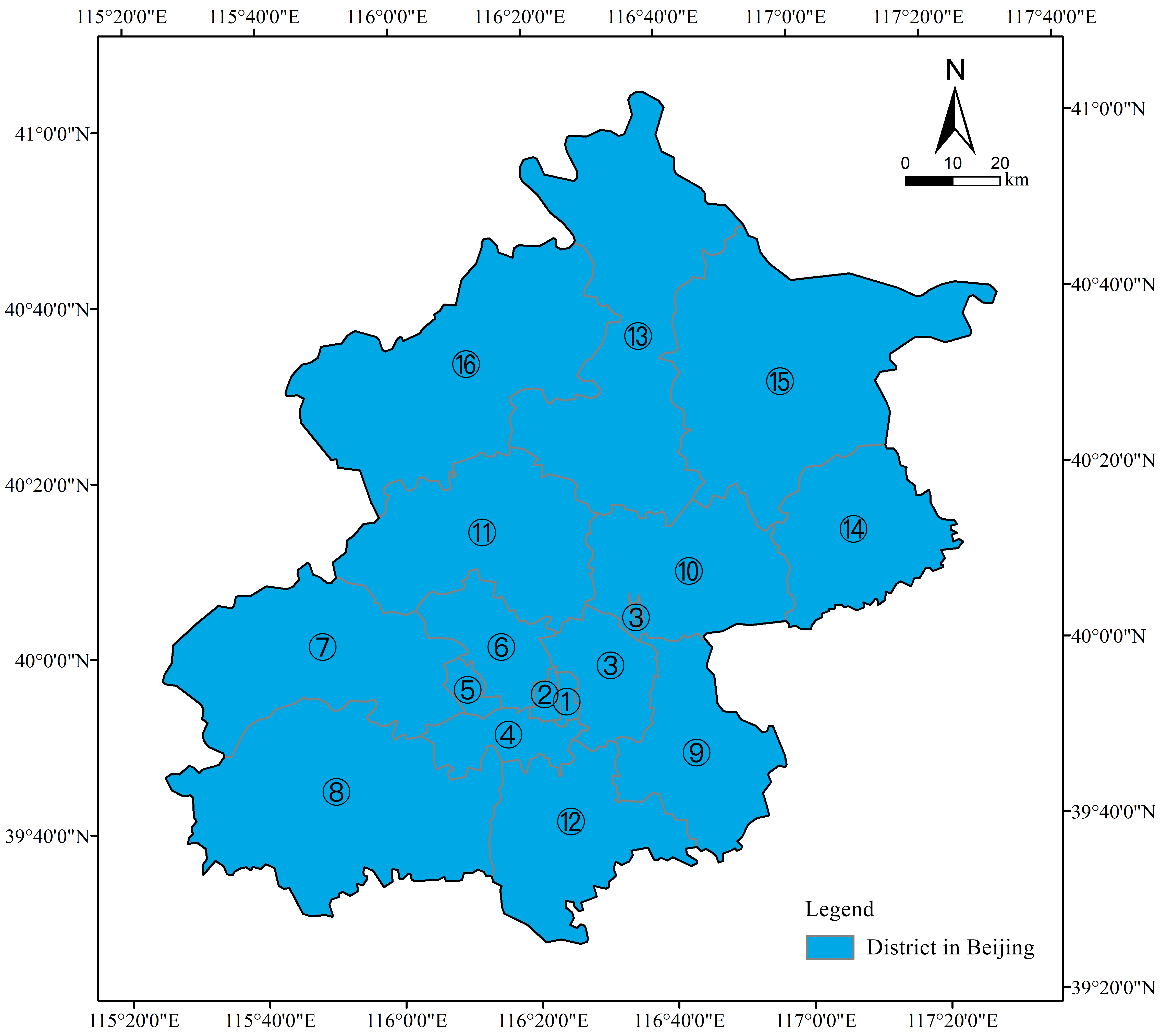


**Fig. S1.**High-resolution administrative map of Beijing. District labels: ① Dongcheng District, ② Xicheng District, ③ Chaoyang District, ④ Fengtai District, ⑤ Shijingshan District, ⑥ Haidian District, ⑦ Mentougou District, ⑧ Fangshan District, ⑨ Tongzhou District, ⑩ Shunyi District, ⑪ Changping District, ⑫ Daxing District, ⑬ Huairou District, ⑭ Pinggu District, ⑮ Miyun District, ⑯ Yanqing District.


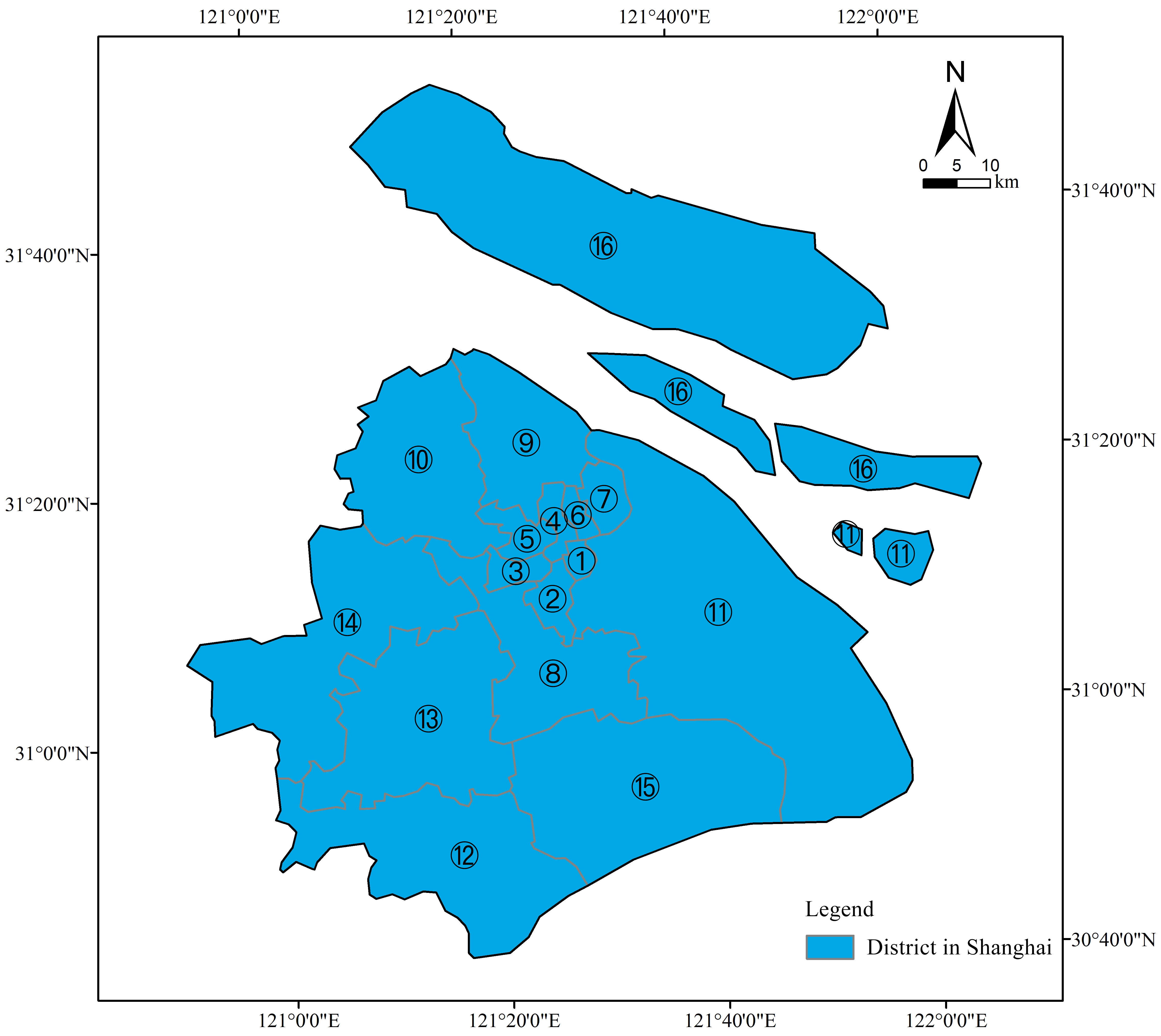


**Fig. S2.** High-resolution administrative map of Shanghai. District labels: ① Huangpu District, ② Xuhui District, ③ Changning District, ④ Jing’an District, ⑤ Putuo District, ⑥ Hongkou District, ⑦ Yangpu District, ⑧ Minhang District, ⑨ Baoshan District, ⑩ Jiading District, ⑪ Pudong New Area, ⑫ Jinshan District, ⑬ Songjiang District, ⑭ Qingpu District, ⑮ Fengxian District, ⑯ Chongming District.


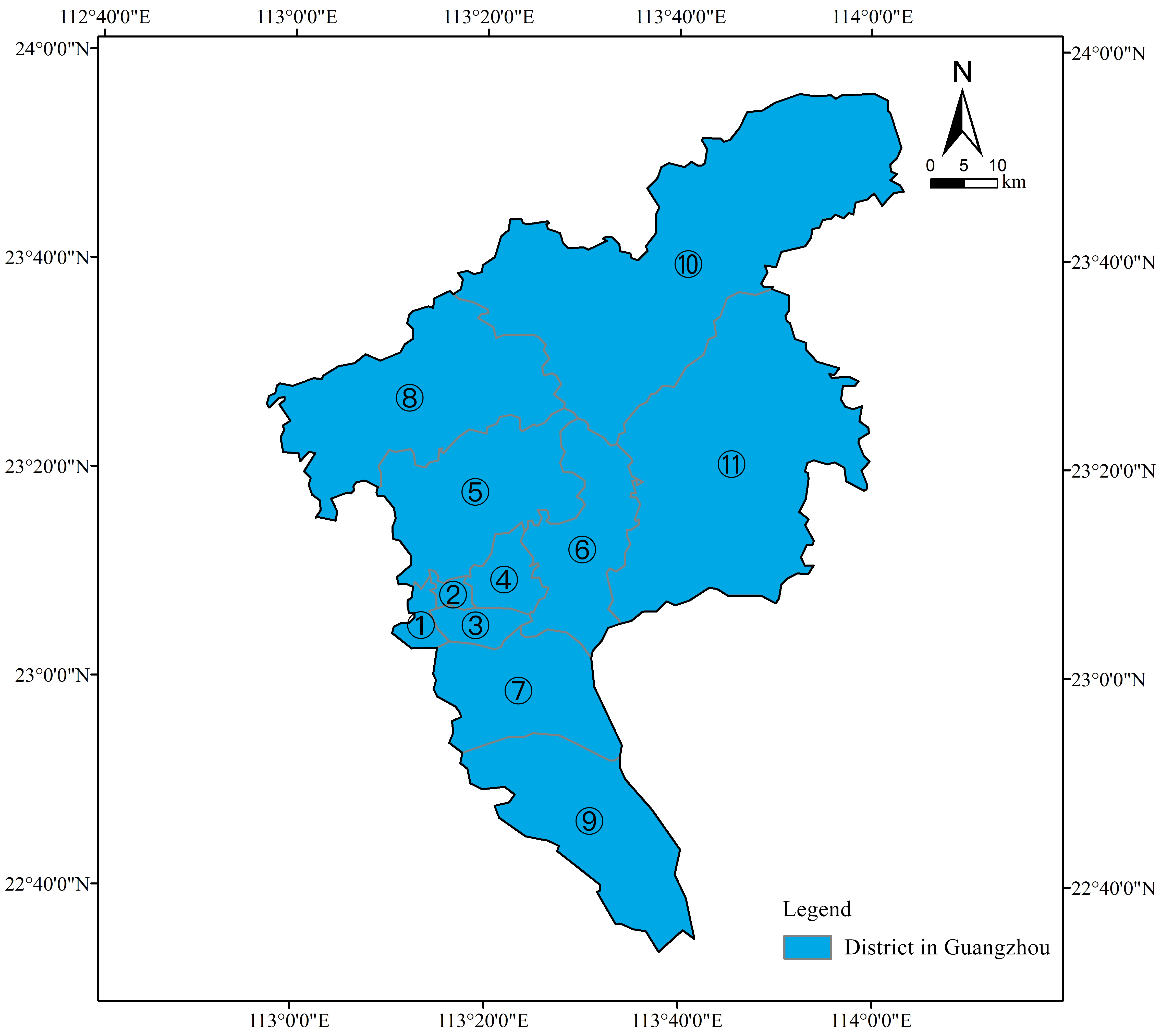


**Fig. S3.** High-resolution administrative map of Guangzhou. District labels: ① Liwan District, ② Yuexiu District, ③ Haizhu District, ④ Tianhe District, ⑤ Baiyun District, ⑥ Huangpu District, ⑦ Panyu District, ⑧ Huadu District, ⑨ Nansha District, ⑩ Conghua District, ⑪ Zengcheng District.


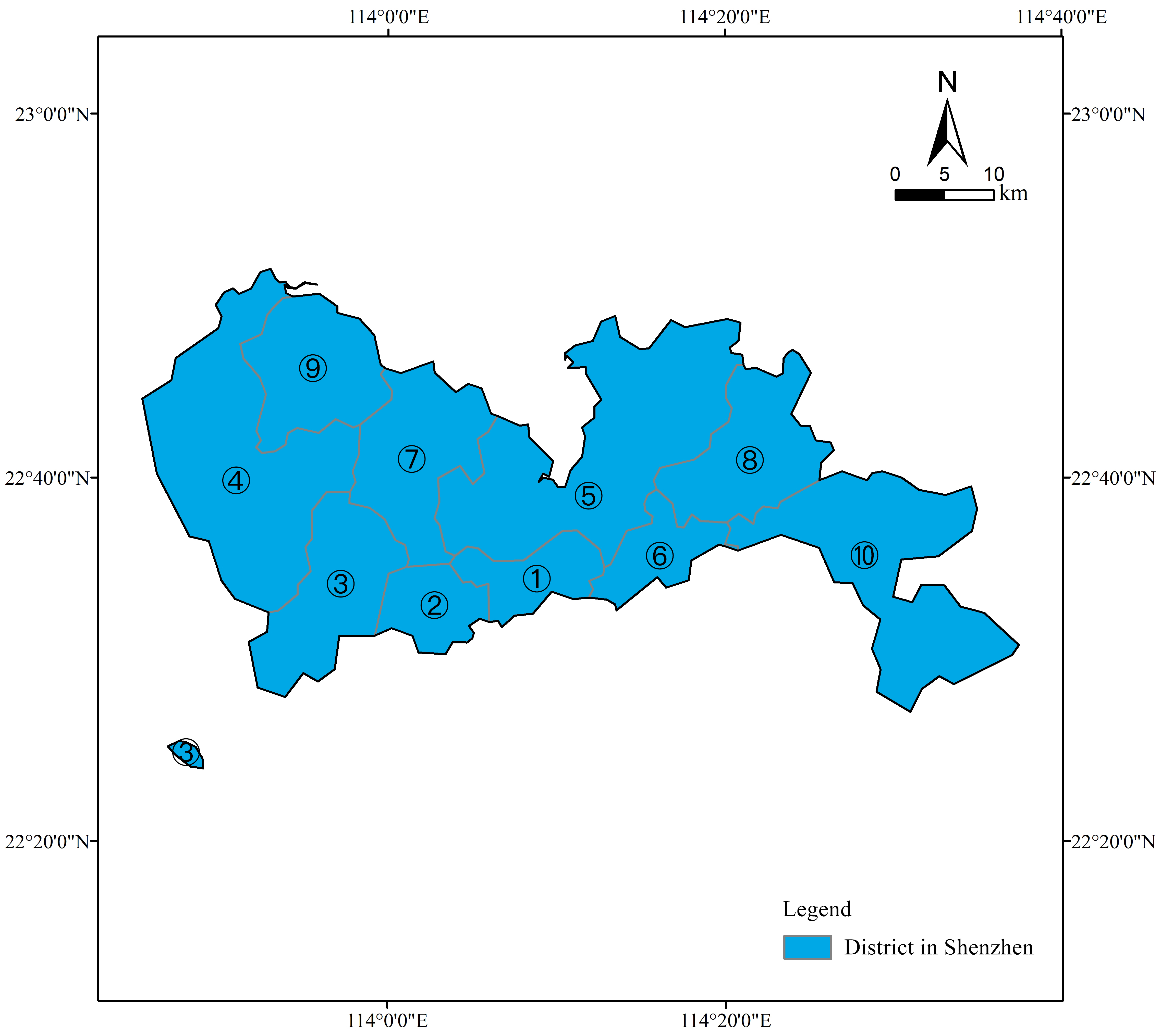


**Fig. S4.** High-resolution administrative map of Shenzhen. District labels: ① Luohu District, ② Futian District, ③ Nanshan District, ④ Bao’an District, ⑤ Longgang District, ⑥ Yantian District, ⑦ Longhua District, ⑧ Pingshan District, ⑨ Guangming District, ⑩ Dapeng New District.

**S2. Detailed Data Processing and Cleaning Steps for AED Locations**

The raw AED location data were obtained via web scraping and contained multiple attributes, including longitude, latitude, device serial number (SN), address, and operational status. To ensure data quality and spatial reliability for subsequent analysis, a five-step cleaning procedure was implemented:

Coordinate Unification: All geographic coordinates were converted to the WGS 1984 coordinate system using the Amap API to ensure consistency for spatial operations.

Spatial Verification: A random sample of AED points in each city was visually cross-referenced with satellite imagery to verify the congruence between their spatial coordinates and the described address in the address field.

Status Filtering: Records with a status field labeled as "Faulty" or equivalent were removed, retaining only devices indicated as operational.

Duplicate Removal (by SN): Exact duplicate records, identified by identical device serial numbers (sn), were deleted.

Spatial Deduplication: Remaining records with identical latitude and longitude (rounded to six decimal places) were considered spatial duplicates, and all but one were removed.

This rigorous cleaning pipeline resulted in a refined dataset of spatially reliable and presumably operational AED locations, forming the basis for all equity analyses presented in the main text.

**S3. Methodological Details of the Entropy Weight Method**

This section elaborates on the entropy weight method applied to determine the objective weights ($W_{SD}$, $W_{C}$, $W_{A}$, $W_{GC}$) for the four equity indicators in Eq. (1) of the main text. The computation proceeds through the following five steps:

Step 1: Indicator orientation alignment

The Gini Coefficient ($GC_{k}$) is a negative indicator (lower values denote greater equity), whereas the Supply-Demand Ration Index ($SD_{k}$), Facility Coverage Index ($C_{k}$), and Accessibility Index ($A_{k}$) are positive indicators. To harmonize their directions, the maximum-value transformation was applied to the negative indicator:

$x_{kj}^{'}=\left\{ \begin{matrix} x_{kj}, & \text{for positive indicators} \\ \max_{1\leq k\leq z}(x_{kj})-x_{kj}, & \text{for negative indicators} \end{matrix} \right.$

S3(1)

where $x_{kj}$ denotes the raw value of subdistrict $k$ for indicator $j$ within a city, and $z$ represents the total number of subdistricts in that city.

Step 2: Min-max normalization

The oriented data were normalized using the min-max method to eliminate dimensional effects, yielding $f_{kj}\in[0,1]$:

$f_{kj}=\frac{x_{kj}^{'}-\min_{1\leq k\leq z}(x_{kj}^{'})}{\max_{1\leq k\leq z}(x_{kj}^{'})-\min_{1\leq k\leq z}(x_{kj}^{'})}$

S3(2)

Step 3: Computing indicator proportions

The proportion $p_{kj}$ of subdistrict $k$ under indicator $j$ was calculated as:

$p_{kj}=\frac{f_{kj}}{\sum_{k=1}^{z} f_{kj}}\text{such that}\sum_{k=1}^{z} p_{kj}=1$

S3(3)

Step 4: Calculating Information Entropy

The information entropy $e_{j}$ for each indicator $j$ was computed using Shannon's formula:

$e_{j}=-\frac{1}{\ln z}\sum_{k=1}^{z} p_{kj}\ln p_{kj}$

S3(4)

*Note:* when $p_{kj}=0$, we define $p_{kj}\ln p_{kj}=0$ to avoid undefined values.

Step 5: Determining final weights

The weight $w_{j}$ for indicator $j$ was derived from its entropy:

$w_{j}=\frac{1-e_{j}}{\sum_{j=1}^{m} (1-e_{j})}\text{such that}\sum_{j=1}^{m} w_{j}=1$

S3(5)

where $m=4$ is the total number of indicators. These weights were calculated independently for each city, resulting in the values of $W_{SD}$, $W_{C}$, $W_{A}$, and $W_{GC}$ used in Eq (1).

The final weights reflect the relative amount of discriminatory information each indicator carries across the subdistricts within a city. A higher weight is assigned to an indicator with greater variability (i.e., lower information entropy).

**S4. Methodological Details of the PCA-GWR Framework**

This section provides the complete computational framework for the Principal Component Analysis combined with Geographically Weighted Regression (PCA-GWR) model referenced in Section 2.3.3 of the main text. The purpose of this two-stage approach is twofold: (1) to address the substantial multicollinearity among the seven original explanatory variables (Table 5, main text) by transforming them into a set of uncorrelated principal components (PCs), and (2) to subsequently model the spatially varying relationships between these PCs and the Comprehensive Equity Index ($I_{k}$) using GWR.

The framework proceeds as follows: Firstly, we present diagnostics confirming the necessity and suitability of PCA for our explanatory variables. Secondly, we detail the sequential steps of PCA (data standardization, component extraction, and interpretation) and GWR (model specification and local estimation).

**S4.1. Suitability and Necessity of PCA for Explanatory Variables**

Prior to conducting the PCA, we assessed the validity of applying this dimension-reduction technique to our dataset. High multicollinearity among predictors not only justifies the use of PCA but is also a prerequisite for its effectiveness. As shown in Table S1, the Variance Inflation Factor (VIF) for several variables—particularly Built-up Area Percentage (BAP) and Road Density (RD)—exceeds the common threshold of 10 across all cities, indicating severe multicollinearity. This confirms the necessity of PCA to create orthogonal predictors for the subsequent regression.

Furthermore, the Kaiser-Meyer-Olkin (KMO) measure of sampling adequacy (all values > 0.6) and statistically significant Bartlett’s tests of sphericity (p < 0.001) verify that the correlations within the dataset are appropriate for reliable component extraction.

**Table S1**

Multicollinearity Diagnostics (VIF) and PCA Suitability Tests for the Seven Explanatory Variables.

| Variable | Beijing | Shanghai | Guangzhou | Shenzhen |
| --- | --- | --- | --- | --- |
| PD | 10.656 | 8.310 | 5.215 | 8.510 |
| GDPPC | 6.460 | 7.833 | 3.314 | 5.107 |
| GDPD | 7.862 | 10.826 | 4.595 | 5.564 |
| MF | 8.403 | 9.730 | 6.322 | 19.622 |
| PV | 7.479 | 9.596 | 7.557 | 14.098 |
| BAP | 16.623 | 22.341 | 19.737 | 49.621 |
| RD | 13.849 | 16.745 | 15.814 | 38.473 |
| KMO | 0.628 | 0.618 | 0.620 | 0.643 |
| Bartlett $\chi^{2}$ (21) | 1908.270^**^ | 1061.328^**^ | 700.990^**^ | 417.762^**^ |

*Note*: “**” indicates p < 0.001.

Having established the need for and suitability of PCA, the following subsections detail its application and the subsequent GWR modeling.

**S4.2. Principal Component Analysis (PCA) Procedure for Dimensionality Reduction**

As detailed in the main text, the first stage of the PCA-GWR framework involves using PCA to transform the seven correlated explanatory variables into a smaller set of uncorrelated principal components (PCs). This process comprised the following five steps (Steps 1-5):

Step 1. Data standardization

Prior to principal component analysis, all explanatory variables were standardized using Z-score normalization to eliminate the influence of differing measurement units and scales. This procedure ensures that each variable has a mean of 0 and a standard deviation of 1. The standardization formula is as follows:

$x_{kq}=\frac{y_{kq}-\bar{y}_{q}}{\sigma_{q}}\left( k=1,2,\cdots,z;q=1,2,\cdots,p \right)$

S4(1)

Where $x_{kq}$ denotes the standardized value of the $q$-th explanatory variable in subdistrict $k$; $y_{kq}$ represents the original value of the $q$-th explanatory variable in subdistrict $k$; $\overline{y}_{q}=\frac{1}{z}\sum_{k=1}^{z} y_{kq}$ is the mean value of the $q$-th explanatory variable across all subdistricts; $\sigma_{q}=\sqrt{\frac{1}{z-1}\sum_{k=1}^{z} (y_{kq}-\overline{y}_{q})^{2}}$ denotes the sample standard deviation of the $q$-th explanatory variable; $z$ represents the total number of subdistricts, and $p$ indicates the total number of explanatory variables.

Step 2. Construction of the standardized data matrix

After standardization, the explanatory variables were assembled into a $z\times p$ standardized data matrix, expressed as:

$\mathbf{X}=\left[ \begin{matrix} x_{11} & x_{12} & \cdots& x_{1p} \\ x_{21} & x_{22} & \cdots& x_{2p} \\ \vdots& \vdots& \ddots& \vdots\\ x_{z1} & x_{z2} & \cdots& x_{zp} \end{matrix} \right]$

S4(2)

where $\mathbf{X}$ denotes the standardized data matrix; $z$ represents the total number of subdistricts, and $p$ indicates the total number of explanatory variables.

Step 3. Eigen decomposition and contribution rate calculation

The correlation matrix of the standardized data matrix $\mathbf{X}$ was first computed as:

$\mathbf{R}=\frac{1}{z-1}\mathbf{X}^{T}\mathbf{X}$

S4(3)

Eigen decomposition was then performed on the correlation matrix $\mathbf{R}$ , $p$ yielding eigenvalues ordered in descending order $\lambda_{1}>\lambda_{2}>\cdots>\lambda_{p}$. Each eigenvalue $\lambda_{m}$ corresponds to an eigenvector $\mathbf{a}_{m}=(a_{m1},a_{m2},\cdots,a_{mp})^{T}$, which represents the loading vector of the $m$ principal component (PC).

The contribution rate of each PC and the cumulative contribution rate are calculated as follows:

$\mu_{m}=\frac{\lambda_{m}}{\sum_{q=1}^{p} \lambda_{q}}$

S4(4)

$\mu_{1:h}=\frac{\sum_{m=1}^{h} \lambda_{m}}{\sum_{q=1}^{p} \lambda_{q}}$

S4(5)

where denotes the contribution rate of the $m$-th PC; $\mu_{1:h}$ represents the cumulative contribution rate of the first $h$ PCs. The criterion for PC extraction is a cumulative contribution rate $\mu_{1:h}\geq80\%$, meaning the first $h$ PCs are retained for subsequent analysis.

Step 4. Determination of the number of principal components and loading matrix

Based on the criterion that the cumulative explained variance is greater than or equal to 80%, the first $h$ PCs were retained. The corresponding eigenvectors were then assembled to form a $p\times h$ loading matrix, expressed as:

$\mathbf{A}=\left[ \begin{matrix} a_{11} & a_{21} & \cdots& a_{h1} \\ a_{12} & a_{22} & \cdots& a_{h2} \\ \vdots& \vdots& \ddots& \vdots\\ a_{1p} & a_{2p} & \cdots& a_{hp} \end{matrix} \right]=(\mathbf{a}_{1},\mathbf{a}_{2},\cdots,\mathbf{a}_{h})$

S4(6)

where $\mathbf{A}$ denotes the $p\times h$ loading matrix; $p$ represents the number of original explanatory variables; and $h$ indicates the number of retained PCs.

Step 5. Calculation of principal component scores

The PC score matrix was obtained by multiplying the standardized data matrix $\mathbf{X}$ with the loading matrix $\mathbf{A}$, yielding a $z\times h$ PC score matrix $\mathbf{F}$:

$\mathbf{F}=\mathbf{XA}$

S4(7)

The element $F_{mk}$ of matrix $\mathbf{F}$ was calculated as:

$F_{mk}=\sum_{q=1}^{p} a_{mq}x_{kq}$

S4(8)

where $F_{mk}$ denotes the score of the $m$-th PC in subdistrict $k$; $a_{mq}$ represents the loading of the $m$-th PC on the $q$-th original variable; and $x_{kq}$ is the standardized value of the $q$-th explanatory variable in subdistrict $k$.

**S4.3. Geographically Weighted Regression (GWR) Modeling with PCA Scores**

In the second stage, the extracted PC scores ($\mathbf{F}$) were used as explanatory variables in a Geographically Weighted Regression (GWR) model to investigate their spatially varying relationships with the Comprehensive Equity Index ($I_{k}$).

Step 6. GWR Model Specification and Estimation

An adaptive bi-square kernel function was used to define the spatial weight matrix, with the optimal bandwidth determined by minimizing the corrected Akaike Information Criterion (AICc) via a golden section search. The local model for each subdistrict $k$ is formulated as:

$I_{k}=\beta_{0}(u_{k},v_{k})+\sum_{m=1}^{h} \beta_{m}(u_{k},v_{k})F_{mk}+\varepsilon_{k}$

S4(9)

where $I_{k}$ denotes the Comprehensive Equity Index of AED allocation in subdistrict $k$; $(u_{k},v_{k})$ represents the spatial centroid coordinates of subdistrict $k$; $\beta_{0}(u_{k},v_{k})$ is the spatially varying intercept; $\beta_{m}(u_{k},v_{k})$ denotes the local regression coefficient of the $m$-th PC in subdistrict $k$; $h$ is the number of retained PCs; $F_{mk}$ is the score of the $m$-th PC in subdistrict $k$; and $\varepsilon_{k}$ represents the random error term.

**S5. Diagnostics and Robustness Checks for the Comprehensive Equity Index (CEI)**

This section provides diagnostic and robustness checks to assess the potential influence of multicollinearity among indicators and the choice of weighting schemes on the construction of the comprehensive equity index (CEI). Rather than assuming full independence among indicators, these checks aim to evaluate whether the observed spatial equity patterns are sensitive to alternative aggregation strategies. First, we assess the degree of multicollinearity among its constituent indicators. Second, we validate the robustness of the index by constructing an alternative version using a different weighting methodology (Principal Component Analysis) and comparing the results.

**S5.1. Multicollinearity Diagnostics for the Four Equity Indicators**

A potential concern when constructing a composite index from multiple indicators is multicollinearity, which could distort the weighting scheme. To assess this, we calculated the Variance Inflation Factor (VIF) for each of the four equity indicators (Supply-demand ration index ($SD_{k}$), Facility coverage index ($C_{k}$), Accessibility index ($A_{k}$), and Gini Coefficient ($GC_{k}$) within each city. A VIF value exceeding 7.5 is often considered indicative of problematic multicollinearity.

**Table S2**

Variance Inflation Factors (VIF) for the Four Constituent Indicators of the Comprehensive Equity Index.

| **Indicator** | **Beijing** | **Shanghai** | **Guangzhou** | **Shenzhen** |
| --- | --- | --- | --- | --- |
| Supply-demand ration index | 1.149 | 1.390 | 1.360 | 1.629 |
| Facility coverage index | 5.860 | 6.015 | 4.973 | 4.935 |
| Accessibility index | 1.221 | 1.165 | 1.393 | 2.089 |
| Gini Coefficient | 5.723 | 6.524 | 5.531 | 4.854 |

As shown in Table S2, all VIF values are below 7, and the maximum value observed is 6.524 (for GC in Shanghai). These results confirm that multicollinearity among the four equity indicators is not severe, and thus does not undermine the validity of the entropy weight method used to aggregate them.

**S5.2. Robustness Check: Constructing an Alternative Index via Principal Component Analysis (PCA)**

To assess the robustness of the Comprehensive Equity Index derived from the Entropy Weight Method (hereafter referred to as EWM-CEI) presented in the main text, we constructed an alternative composite index using Principal Component Analysis (hereafter referred to as PCA-CEI). This approach employs a fundamentally different objective weighting scheme—based on the variance-covariance structure of the indicators—in contrast to the information-entropy-based logic of the Entropy Weight Method. Comparing the results from these two methodologies tests whether the spatial equity rankings are sensitive to the specific choice of weighting algorithm, thereby evaluating the robustness of the composite index.

It is critical to distinguish the application of PCA in this section from its use in Section S4. In Section S4, PCA was employed as a necessary dimensionality reduction tool to address severe multicollinearity among the seven original explanatory variables for regression modeling. Here, in the absence of severe multicollinearity among the four equity indicators (as confirmed in Section S5.1), PCA is used solely as an alternative objective weighting method for the purpose of robustness validation. The Spearman's rank correlation coefficient between the EWM-CEI and PCA-CEI scores was calculated to quantitatively evaluate the consistency of the spatial equity rankings produced by the two distinct composite indices.

**S5.2.1. Method: PCA-based Weighting Procedure for the Equity Indicators**

The procedure for constructing the PCA-CEI comprised the following seven sequential steps:

Step 1. Indicator orientation normalization

Prior to analysis, all negative indicators were normalized to ensure consistent orientation (higher values indicating better performance). The transformation rule is given by:

$x_{kj}^{'}=\left\{ \begin{matrix} x_{kj}, & \text{for positive indicators} \\ \max_{1\leq k\leq z}(x_{kj})-x_{kj}, & \text{for negative indicators} \end{matrix} \right.$

S5(1)

where $x_{kj}$ denotes the original value of the $j$-th indicator for subdistrict $k$; $z$ is the total number of subdistricts; and $x_{kj}^{'}$ represents the oriented value where all indicators are transformed to positive direction.

Step 2. Data standardization

All explanatory variables were standardized using Z-score normalization to eliminate dimensional and scale differences, ensuring each variable has zero mean and unit variance:

$x_{kj}=\frac{x_{kj}^{'}-\overline{x}_{j}^{'}}{s_{j}},k=1,2,\ldots,z;j=1,2,\ldots,p$

S5(2)

where $x_{kj}$ denotes the standardized value of the $j$-th indicator for subdistrict $k$; $x_{kj}^{'}$ is the oriented value from Step 0; $\bar{x}_{j}^{'}=\frac{1}{z}\sum_{k=1}^{z}x_{kj}^{'}$ represents the sample mean of indicator $j$; $s_{j}=\sqrt{\frac{1}{z-1}\sum_{k=1}^{z} \left( x_{kj}^{'} - \overline{x}_{j}^{'} \right)^{2}}$ is the sample standard deviation of indicator $j$; $z$ is the total number of subdistricts; and $p$ is the total number of indicators.

Step 3. Construction of the standardized data matrix

The standardized values were assembled into a $z\times p$ matrix:

$\mathbf{X}=\left[ \begin{matrix} x_{11} & x_{12} & \cdots& x_{1p} \\ x_{21} & x_{22} & \cdots& x_{2p} \\ \vdots& \vdots& \ddots& \vdots\\ x_{z1} & x_{z2} & \cdots& x_{zp} \end{matrix} \right]$

S5(3)

where $\mathbf{X}$ denotes the standardized data matrix.

Step 4. Correlation matrix and eigen decomposition

The correlation matrix of $\mathbf{X}$ was computed as:

$\mathbf{R}=\frac{1}{z-1}\mathbf{X}^{\top}\mathbf{X}$

S5(4)

Eigen decomposition of $\mathbf{R}$ yields eigenvalues in descending order $\lambda_{1}>\lambda_{2}>\cdots>\lambda_{p}$ and corresponding eigenvectors $\mathbf{a}_{m}=(a_{m1},a_{m2},\cdots,a_{mp})^{T}$ for $m=1,2,\ldots,p$.

The contribution rate of the $m$-th principal component and the cumulative contribution rate are:

$\mu_{m}=\frac{\lambda_{m}}{\sum_{q=1}^{p} \lambda_{q}}$

S5(5)

$\mu_{\left( 1 : h \right)}=\frac{\sum_{m=1}^{h} \lambda_{m}}{\sum_{q=1}^{p} \lambda_{q}}$

S5(6)

where $\mu_{m}$ denotes the individual contribution rate and $\mu_{1:h}$ represents the cumulative contribution rate of the first $h$ principal components. The extraction criterion requires $\mu_{1:h}\geq80\%$.

Step 5. Principal component retention and loading matrix

Based on the 80% cumulative variance criterion, the first $h$ principal components were retained. The corresponding eigenvectors form the $p\times h$ loading matrix:

$\mathbf{A}=\left[ \begin{matrix} a_{11} & a_{21} & \cdots& a_{h1} \\ a_{12} & a_{22} & \cdots& a_{h2} \\ \vdots& \vdots& \ddots& \vdots\\ a_{1p} & a_{2p} & \cdots& a_{hp} \end{matrix} \right]=(\mathbf{a}_{1},\mathbf{a}_{2},\ldots,\mathbf{a}_{h})$

S5(7)

where $a_{mj}$ represents the loading of the $m$-th principal component on the $j$-th original indicator.

Step 6. Calculation of indicator weights

The initial weight for each indicator was derived by weighting squared loadings by variance contribution rates:

$w_{j}^{*}=\sum_{m=1}^{h} a_{mj}^{2}\cdot\frac{\lambda_{m}}{\sum_{q=1}^{p} \lambda_{q}},j=1,2,\ldots,p$

S5(8)

The final normalized weights were obtained via:

$w_{j}=\frac{w_{j}^{*}}{\sum_{t=1}^{p} w_{t}^{*}}, \text{with; }\sum_{j=1}^{p} w_{j}=1$

S5(9)

where $w_{j}$ denotes the ultimate weight of the $j$-th indicator in the composite evaluation system.

Step 7. Composite score synthesis

The PCA-weighted composite score for subdistrict $k$ is calculated as:

$S_{k}=\sum_{j=1}^{p} w_{j}x_{kj},k=1,2,\ldots,z$

S5(10)

where $S_{k}$ represents the comprehensive evaluation score for subdistrict $k$, integrating all indicators according to their PCA-derived weights. Higher $S_{k}$ values indicate better performance in AED allocation equity.

**S5.2.2. Results: PCA Output and Derived Weights**

PCA was performed separately on the four oriented and Z-score standardized equity indicators for each city. Table S3 presents the eigenvalues and variance explained by the extracted principal components. Based on the criterion of cumulative explained variance exceeding 80%, the first two principal components (PC1 and PC2) were retained for all cities, collectively explaining over 95% of the total variance in each case.

**Table S3**

Results of Principal Component Analysis on the Four Equity Indicators.

| City | Component | Eigenvalue | Proportion of Variance (%) | Cumulative Proportion of Variance (%) |
| --- | --- | --- | --- | --- |
| Beijing | PC1 | 2.202 | 54.88% | 54.88% |
|  | PC2 | 1.681 | 41.89% | 96.77% |
| Shanghai | PC1 | 2.818 | 70.13% | 70.13% |
|  | PC2 | 1.104 | 27.47% | 97.60% |
| Guangzhou | PC1 | 2.085 | 51.82% | 51.82% |
|  | PC2 | 1.886 | 46.86% | 98.68% |
| Shenzhen | PC1 | 2.015 | 49.69% | 49.69% |
|  | PC2 | 1.844 | 45.49% | 95.18% |

The PCA-derived weights for each original indicator were then calculated from the squared loadings of the retained components, weighted by their respective variance contributions (as detailed in Step 6 of Section S5.2.1). The final weights, presented in Table S4, are remarkably balanced across the four indicators for each city. This contrasts with the more variable weights produced by the entropy method (Table 4 in the main text), highlighting the different theoretical foundations of the two methods.

**Table S4**

PCA-derived Weights for the Four Equity Indicators.

| **Dimension** | **Indicator** | **City** | **Weight** |
| --- | --- | --- | --- |
| Resource allocation equity | Supply-demand ration index | Beijing | 24.86% |
|  |  | Shanghai | 24.80% |
|  |  | Guangzhou | 25.17% |
|  |  | Shenzhen | 25.20% |
| Spatial coverage equity | Facility coverage index | Beijing | 25.12% |
|  |  | Shanghai | 25.20% |
|  |  | Guangzhou | 24.83% |
|  |  | Shenzhen | 24.80% |
| Opportunity accessibility equity | Accessibility index | Beijing | 24.88% |
|  |  | Shanghai | 24.80% |
|  |  | Guangzhou | 25.17% |
|  |  | Shenzhen | 25.20% |
| Spatial distribution equity | Gini Coefficient | Beijing | 25.14% |
|  |  | Shanghai | 25.20% |
|  |  | Guangzhou | 24.83% |
|  |  | Shenzhen | 24.80% |

**S5.2.3. Correlation between EWM-CEI and PCA-CEI**

The PCA-weighted composite score (PCA-CEI) was calculated for each subdistrict and compared with the original EWM-CEI using Spearman's rank correlation analysis. As shown in Fig. S5, a very strong positive correlation (Spearman's $\rho>0.9$) was observed for all four cities.

Conclusion of the Robustness Check: The low VIF values among the four base indicators (Table S2) and the exceptionally high correlation between the EWM-CEI and PCA-CEI (Fig. S5) jointly demonstrate that the Comprehensive Equity Index constructed in this study is robust. Its outcomes are not sensitive to the specific choice of objective weighting methodology (entropy vs. PCA), and the relative spatial equity rankings of subdistricts remain stable.


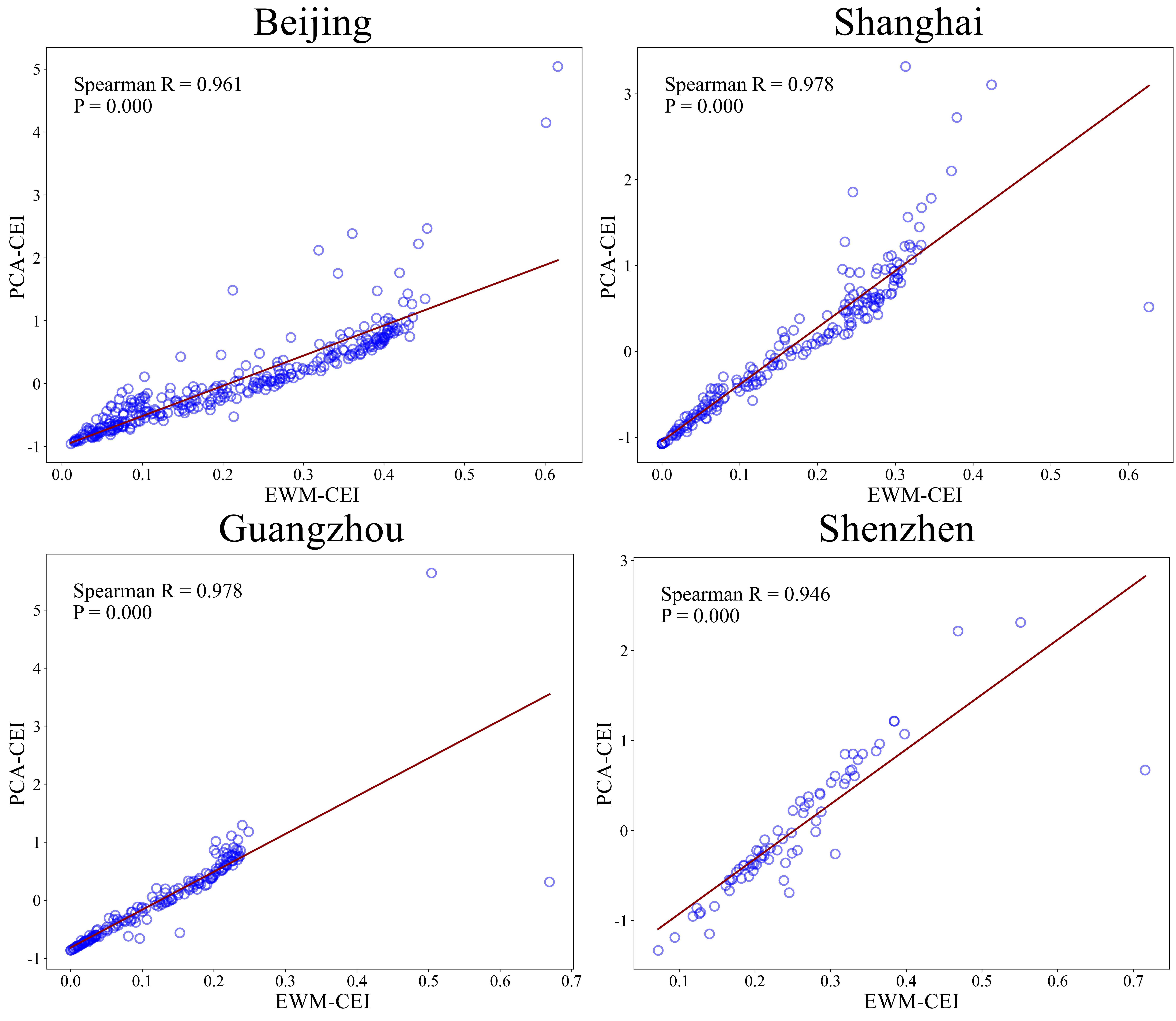


**Fig. S5.** Robustness check: Scatter plots of the Entropy Weight Method-based CEI (EWM-CEI, x-axis) against the Principal Component Analysis-based CEI (PCA-CEI, y-axis). The reported Spearman's rank correlation coefficient ($\rho>0.9$)) for each city indicates a near-perfect monotonic relationship between the rankings produced by the two different composite index methods.

**S6. Summary of Local Coefficient Significance for PCA-GWR Models**

To complement the model fit statistics presented in Table 7 of the main text, this section provides a detailed summary of the statistical significance of the local regression coefficients from the PCA-GWR models. For each city and each principal component, we calculated the percentage of subdistricts where the local coefficient was statistically significant at the p < 0.05 level. These results are summarized in Table S5.

**Table S5**

Summary of Local Coefficient Significance for PCA-GWR Models.

| City | Component | % of Subdistricts with p < 0.05 | % Significant & Positive | % Significant & Negative |
| --- | --- | --- | --- | --- |
| Beijing | DU | 87.61% | 87.61% | 0.00% |
|  | PSFP | 30.82% | 0.00% | 30.82% |
|  | WPDT | 55.59% | 2.42% | 53.17% |
| Shanghai | DU | 67.29% | 64.02% | 3.27% |
|  | PSFP | 24.77% | 3.74% | 21.03% |
|  | WPDT | 13.08% | 10.75% | 2.34% |
| Guangzhou | DU | 92.94% | 92.94% | 0.00% |
|  | PSFP | 0.59% | 0.59% | 0.00% |
|  | WPDT | 5.88% | 0.00% | 5.88% |
| Shenzhen | DU | 75.68% | 75.68% | 0.00% |
|  | PSFP | 74.32% | 0.00% | 74.32% |
|  | WPDT | 31.08% | 31.08% | 0.00% |

Note: Percentages are calculated based on the total number of subdistricts in each city. DU = Degree of Urbanization Component; PSFP = Public Service Facility Provision Component; WPDT = Wealth-Population Density Trade-off Component.
